# Die Pellagra – vor 250 Jahren im Kaisertum Österreich erstmals beschrieben, wurde sie zu einer lebensbedrohenden Endemie in einigen Provinzen

**DOI:** 10.1007/s00508-021-01840-z

**Published:** 2021-04-21

**Authors:** Heinz Flamm

**Affiliations:** 1 Martinstraße 7, 3400 Klosterneuburg, Österreich; 2 Medizinische Universität Wien, Wien, Österreich

**Keywords:** „D-Trias“ (Dermatitis – Diarrhoe – Demenz) und Tod, Mais-Ernährung, Bact. maïdis, Zeïsten, Toxikozeïsten, „Disease of the four D’s“ (Dermatitis – Diarrhea – Dementia – Death), Nutrition with maize, Bact. maïdis, Zeïsts, Toxikozeïsts

## Abstract

Die Pellagra ist charakterisiert durch die Trias „Dermatitis – Diarrhoe – Demenz“, gefolgt vom Tod. Verschiedene Ursachen wurden im Laufe zweier Jahrhunderte diskutiert. Anfänglich hielt man starke Sonnenbestrahlung als Ursache von Veränderungen der Strukturen des Körpers. Die „Zeïsten“ waren der Meinung, der Mais als Hauptnahrungsmittel sei die Ursache der Pellagra, die also eine Mangelkrankheit sei. Dagegen meinten die „Toxikozeïsten“, in unreifem Mais oder in zwar reifem jedoch nicht gut getrocknetem oder in schlecht gelagertem Mais oder in schlecht aufbewahrtem Maismehl und ungenügend gebackenem Maisbrot könnten durch harmlose Bakterien und Schimmelpilze giftige, pellagrogene Produkte gebildet werden. Andere Untersucher sahen die Pellagra als Ergebnis von allergischen Reaktionen. Klärung brachten Goldbergers Selbstversuche 1916 und schließlich 1937 der Nachweis Elvehjems des Mangels von Niacin bei Maisernährung.

Im Kaisertum Österreich bzw. (ab 1867) in Österreich-Ungarn wurde die Pellagra in den k.k. Provinzen Küstenland, Tirol und Bukowina wie auch in Ungarn festgestellt und ab verschiedenen Zeiten bekämpft. Noch im Glauben der Schädlichkeit von verdorbenem Mais in der armen Bevölkerung wurden in Landesgesetzen von Tirol 1904, Görz und Gradiska (Küstenland) 1909 und der Bukowina 1911 umfängliche Maßnahmen verlangt. So sollten z. B. zur geregelten Versorgung der Bevölkerung Speisehäuser, Maistrockenöfen, -lagerhäuser und -magazine sowie öffentliche Brotbäckereien eingerichtet werden. Zur Behandlung Pellagrakranker waren Pellagrosarien und Notspitäler zu errichten, die Zahl niedergelassener Ärzte war zu erhöhen. Als wichtig wurde auch die Aufklärung der Bevölkerung verlangt. Ein Pellagrafonds sollte die Maßnahmen unterstützen. Als beratendes und begutachtendes Organ wird eine Pellagrakommission unter Vorsitz des Statthalters mit zwölf in ihrer Funktion festgelegten Mitgliedern eingesetzt.

## Summary

Pellagra is characterized by “dermatitis – diarrhea – dementia – death”. Various causes were discussed over the course of two centuries. The initial presumption was that the sun caused changes in exposed areas of the body. The “Zeïsts” blamed the maize (corn), which forms the main constituent in the diet of poor peoples, for being an insufficient nutrient and thus causing the pellagra in such indigent populations. The “Toxikozeïsts”, however, regarded toxins produced by innocuous bacteria and fungi in unripe or in ripe but badly stored maize or in maize flour or in poorly baked maize bread as the cause of pellagra. Pellagra as an allergic disease was also discussed. Self-experiments of Goldberger’s group in 1916 and finally Elvehjem’s detection of niacin deficiency in maize in 1937 solved the problem.

In the Austrian empire and (from 1867 on) in the Austro-Hungarian monarchy, pellagra was diagnosed and combated in the provinces of Küstenland, Tirolia and Bukovina and in Hungary. Originally believing in the noxiousness of maize in the poor population, extensive measures were planned and partially executed. Primarily measures for providing salubrious maize products were planned for the population, such as public bakeries and eating houses, kilns and storage houses for maize. For the treatment of pellagra patients, so-called pellagrosaria and auxiliary hospitals were established and the number of general practitioners was increased. It was also important to educate the population about preventing pellagra by consuming proper food. Pellagra funds to sustain the measures were established. In the provinces, pellagra commissions, chaired by the governor and consisting of twelve experts of the relevant medical branches, were appointed as an advisory and expert body.

**Figure 1:**
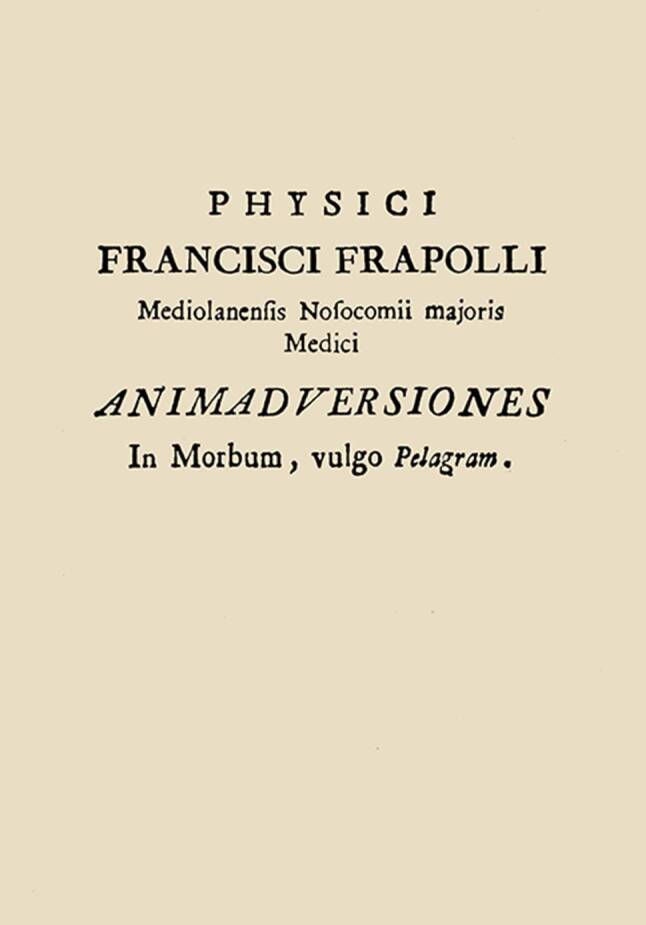

Im September 1771 veröffentlichte Francesco Frapolli (1738–1773), der ärztliche Direktor des Großen Spitals in Mailand, der Hauptstadt der seit 1714 wieder österreichischen Lombardei, seine „Animadversiones In Morbum, vulgo Pelagram“ [1]. In diesen „Beobachtungen einer im Volksmund Pelagra genannten Krankheit“ beschrieb er eine „neue“ Krankheit bei Patienten aus seiner Region Insubria [*Gebiet der Lombardei um Mailand, in dem im Altertum die keltischen Insubrier siedelten*] (Abb. 1).

Frapolli stellte sich einige Fragen, an erster Stelle ob die Pelagra [*sic!*] eine neue Krankheit ist. Da hegte er einen leichten Zweifel, ob nämlich der vor 200 Jahren, im März 1578, in einer Anordnung seines Spitals für die Versorgung bestimmter Kranker verwendete Terminus Pellerella nicht doch bereits die Pelagra betraf. Wie aber auch 1780 Michele Gherardini (1752–1825; [2]) bestätigte, bezeichnete Pellerella eine genital übertragene Krankheit. Dieser Autor weist auch darauf hin, dass etliche andere Haar- und Hautkrankheiten mit den Silben Pel (für Pelo = Haar) oder Pell (für Pelle = Haut) beginnen, wie Pelade, Pelarola oder Pelatina und das [*sic! Neutrum!*] Pellagra. In der umfänglichen Übersicht des „Handbuchs der historisch-geographischen Pathologie“ [3] von August Hirsch (1817–1894) wird die 1762 posthum erschienene Publikation des Leibarztes von Kaiser Ferdinand VI., des Katalanen Gaspar Casal (1680–1759), als erste Nachricht über Pellagra angegeben [4]. Gherardini konnte auch diese als Lepra asturica, Rose delle Asturie oder Mal de la Rosa bezeichnete Krankheit als different von der Pellagra erkennen. Wenn dies stimmt, kann man sagen, dass Frapolli in Mailand die „neue“ Krankheit, die Pellagra, als Erster beschrieben hat.

Diese Krankheit wurde bald unter vielen lokalen Namen bekannt. Diese bezogen sich entweder auf die Hauterscheinungen wie Dermagra *[üble Haut*], Rose delle Asturie, Lepra asturica, Lepra italica, Mal rosso [*Rotes Übel*], Erythema endemicum, Porpora rossa, Risipola lombarda [*Lombardische Wundrose*], Eczema lombardo, Cattivo male [*Gefährliches Übel*], oder auf vermeintliche Ursachen wie Mal del sole [*Übel der Sonne*], Malattia dell’insolatio di primavera [*Frühlings-Insolationskrankheit*], Scottatura di sole [*Sonnen-Brandwunde*], Maidismus, Raphania maisitica [*rasch auftretende Mais-Krankheit*] und Malattia della miseria [*Krankheit der Armut*].

Francesco Frapolli geht von seinen eigenen Beobachtungen aus. Dabei beschäftigte ihn die wichtige Frage, ob die Pelagra infektiös ist. Aus eigener Erfahrung in ländlichen Familien weiß Frapolli, dass die Pelagra weder ad distans, noch per contactum übertragbar ist. Den Beweis fand er durch seine Feststellung, dass die Krankheit nicht auf Gesunde übertragen wurde, die im Krankenhaus viele Tage mit an bestätigter Pelagra Leidenden im selben Bett lagen.

Frapolli schildert ausführlich seine Beobachtungen an seinen Patienten. Als Ursache der Pelagra meinte er, so wie auch die Landbevölkerung in der Insubria glaubte, die im Frühjahr beginnende und sich dann steigernde Sonnenbestrahlung schädige die Haut. Auch die Remissionen und das Wiederauftreten kleinerer oder größerer Hautaffektionen wären Folgen der Insolation. Die Sonne mit ihren heißen vibrierenden Strahlen quält und beunruhigt stark die Körper der Bauern; die Kanäle der Haut werden gleichmäßig auseinander gespannt, die ausdünstenden Hautporen stark erweitert, die transpirable Materie fließt durch die Kraft des Herzens und den Antrieb der Gefäße zusammen, sodass sie gemischt ausfließt. Was kann so die Fülle der Säfte abführen? Ob nicht ein ziemlich wichtiger Teil der Transpiration entkräftet wird? Was wenn die fortgesetzte Aktion der Sonne die eigenartige Anordnung der Hautporen gänzlich verstopft und die Haut runzelig und schwielig wird, dann gibt es in den betroffenen Teilen kein Schwitzen; daher keine Bestätigung der Beeinträchtigung. Das sind die größeren und kleineren Effekte der Pelagra, neben größeren und kleineren Hautveränderungen. Also zusammenfassend ist die Krankheitsursache selbstverständlich die Insolation.

Frapolli fand im ersten Krankheitsstadium eine Rötung der Haut an Händen, Füßen und anderen Körperteilen, die der Sonne ausgesetzt sind. Ein Pruritus folgt und endlich schuppt die Haut. Sie wird runzelig, schwielig und rissig. Als Vorkehrung schützen die Bauern Hände, Arme, Beine und den Kopf gegen die Sonnenbestrahlung. Baden und zumindest das Waschen der Extremitäten werden als nützlich angesehen.

Mit Fortschreiten der Pelagra beginnen nach Frapollis Auffassung die Fasern der Eingeweide gewissermaßen zu erschlaffen. Die Ärzte beobachten ernsthaft eine leichte Änderung in der Erkrankung, eine Diarrhoe kommt dazu. Selbst vorsichtig zurückgehaltene Purganzien, vorwiegend aus Vegetabilien, wie Mus aus Tamarinden [*exotische Leguminosen*], Mixtura stomatica Riveri [*Kaliumkarbonat in Weinsäure*] und andere gleicher Art versagen. Rektal wird das [*eisenhaltige*] Clystirum chalibeati gegeben. Als Getränk, auch wenn der Durst fehlt, soll der Kranke nüchtern verdünnten Wein trinken; denn unverdünnt erheitert er die geschwächten Bauern; wenn der Patient wirklich durstet, trinkt er Molke mit Tamarinden-Mus, Emulsio nitrata oder Zitronenwasser. Das Verabreichte muss bei bestätigter Pelagra gänzlich verdünnt sein, damit die Eingeweide, die gestärkt werden müssen, nicht zu sehr entkräftet werden, was die Krankheit rasch verschlechtert. Das Volk nimmt Herz-Mixturen, Kräftigungsmittel und Hirn-Mittel. Bei Plethora hilft einmaliger Blutentzug, bei starker Manie solcher aus der Vena jugularis. In diesen Zeiten erzeugen Blutegel Wunder. Die allgemeine körperliche Schwäche wird durch Baden oder zumindest durch häufiges partielles Abwaschen oder Umschläge mit erweichenden Dekokten in Molke bekämpft; unterdessen werden auch kräftigende Medizinen verabreicht.

Im zweiten Stadium der Pelagra findet Frapolli die Kranken noch fieberfrei, aber es treten Kopfschmerzen, Furcht, Traurigkeit, Schlaflosigkeit, Vergesslichkeit, Dummheit, hypochondrische Delirien und Manie auf. Durchfall und Kraftverlust werden stärker und schließlich verliert der Patient die Beweglichkeit, am meisten des Kreuzes und der Beine. Zeichen des Fortschreitens der Krankheit sind dann Fieber, komaartige Erscheinungen, dünnflüssige Durchfälle, größte Abmagerung, größte geistige Schwäche und Widerspenstigkeit, und dann folgt der Tod. Alle diese Zeichen der Pelagra werden immer beobachtet, und verschiedene außerordentliche Symptome und Komplikationen, wie etwa Hydropsie [*Wassersucht*], können dazukommen. Bei der Behandlung ist das Wichtigste, die Hautporen für die Exhalation zu öffnen, damit die Transpiration freier wird. Dies erreicht man weiterhin durch das Bad und verstärkt durch Abreibungen der Extremitäten und besonders des Körpers mit rauen wollenen Tüchern. Phlebotomie bei dringlicher Plethora ist der höchste Rat. Auch Blutegel sind wirksam. Auf dem Hinterkopf applizierte blasenziehende Mittel helfen bestens. Einnahme von 8 Unzen Aqua Thaedae [?] zweimal am Tag, jedenfalls Wein und Ähnliches sind zu empfehlen. Als Diät eignen sich mehlige Vegetabilien, nicht aber Fleisch. Der Pelagröse soll einige Tage lang verdünnte Getränke zu sich nehmen, vorwiegend Molke mit Blättern und Antiskorbutmitteln.

Bald kann die Pelagra in ein aussichtsloses Stadium kommen. Darüber „Worte zu machen ist überflüssig; in diesem Krankheitsstadium gibt es keine Reaktion, und die leichenartige Verwesung hat den Körper fest erfasst, & die Herzenskraft hält das Leben noch eine Weile aufrecht, wie ich [*Frapolli*] mehrfach bemerkt habe“.

## Pellagra – die „D-Trias“

**Figure 2:**
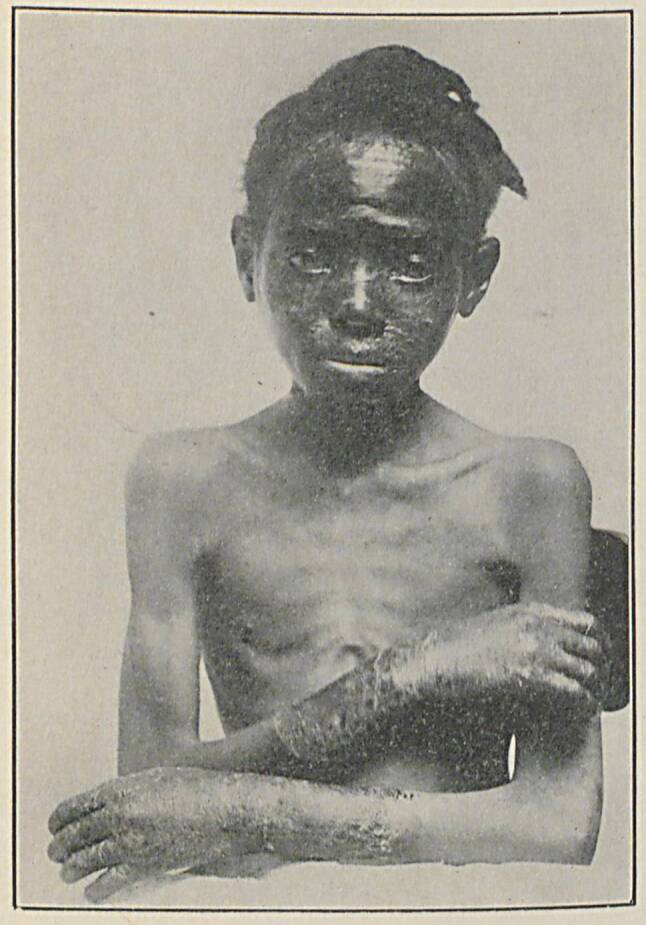

In den Beschreibungen des 19. Jahrhunderts wird die Pellagra durch die „D-Trias“ charakterisiert, nämlich Dermatitis – Diarrhoe – Demenz; sie hat auch den Namen „Disease of the four D’s“, also die drei genannten D‑D-D + Death [Tod]. Nach allgemeinen prodromalen Symptomen wie leichte psychische und nervale Erscheinungen, entwickelt sich eine Dermatitis mit scharf begrenzten pigmentierten Erythemen mit folgenden pigmentierten Hyperkeratosen an den dem Licht oder ständigem Druck ausgesetzten Stellen (Abb. 2 und 3), dann treten wässrig-schleimige Durchfälle und motorische, sensible und trophische Störungen, bei Hirnbeteiligung Psychosen und Demenz auf. Die Krankheit kann viele Jahre bestehen und schließlich zum Tod führen, manchmal auch durch Suizid in delirösem Zustand [5–7].

**Figure 3:**
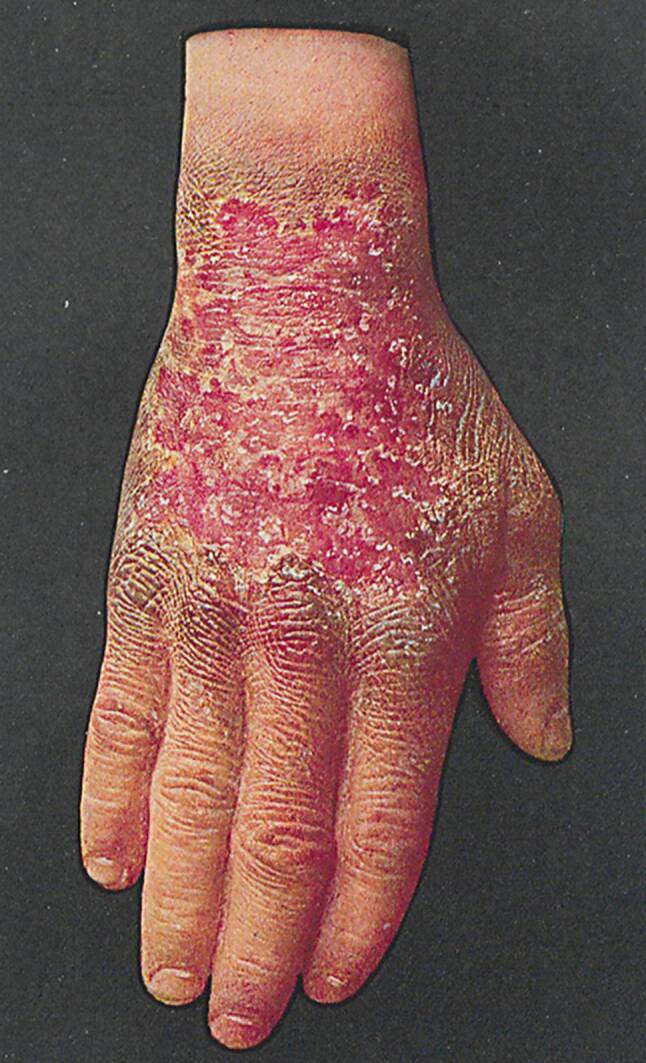

Zweifel an der vom Volk geglaubten Ursache der Pelagra ergaben für Frapolli bei der Beobachtung und Behandlung seiner Patienten verschiedene Fragen: „Ist wirklich die Insolation [*Sonnenbestrahlung*] die einzige Ursache? Genügt sie aber jederzeit zur Erzeugung der Krankheit und gibt es nicht andere zwingende Gründe, die aus sich heraus die Pelagra erzeugen? Warum werden schwitzende Schmiede, im Gegensatz zu schwitzenden Bauern, nicht pelagrös? Warum werden so viele arme Leute, die schlechte Nahrung zu sich nehmen, am wenigsten ergriffen? Warum werden in Zeiten der Teuerung nicht alle Menschen pelagrös? Warum werden auf dem Lande, nicht aber in den Städten, die Leute von der Pelagra erfasst?“

Der Nächste, der sich in der Lombardei mit der Pellagra befasste, war Michele Gherardini (1752–1825; [2]). Er interessierte sich, ob die Pellagra nicht schon früher aufgetreten ist. So konnte er von vielen seiner Patienten erfahren, dass auch deren Voreltern „il mal rosso“ [*in der deutschen Übersetzung von 1792 als „die Rose“ und „Rothlauf“ bezeichnet*] an verschiedenen Körperteilen gehabt hätten, „und sie nannten das Uebel erst alsdann Pellagra, wann es auf den höchsten Grad gestiegen war“. „Was die Benennung Pellagra [*pellis (lat.)* *=* *Haut, agrestis (lat.)* *=* *wild, ländlich*] anbetrifft“, so glaubte Gherardini, „dass die Landleute selbst die Krankheit mit diesem ganz treffenden und charakteristischen Namen benannt haben, auf eben die Art wie der gemeine Mann zu Plinii Zeiten den Kleyenaussaz [*Kleie-Aussatz*] Mentagra nannte (der sonst Lichen hieß), weil er hauptsächlich das Kinn [= *mentum (lat.)*] mit heßlichen kleyenartigen Schuppen einnahm“. „Es ist demnach das [*sic!*] Pellagra eine in (unserem) Insubrien endemische Krankheit“, die nach Gherardinis „Wissen bis jetzt noch nirgends bekannt ist als in unserem Insubrien, wo es, ohne irgend einen vorzugsweise vor dem anderen zu befallen, sich ohne Unterschied bald hier bald dort ausbreitet, und sich nur mit größerer Bösartigkeit da zeigt, wo man eine dünnere und trockenere Luft einathmet“ [2].

Gheradini beschäftigt sich mit der möglichen Ursache der Pellagra, da nach seiner Überzeugung die Sonnenbestrahlung allein nicht genügt. Es erschien ihm aber unwahrscheinlich, dass kleinste Tierchen [*wie sie Antoni van Leeuwenhoek (1632–1723) im Jahre 1675 beschrieben hat*] die Haut befallen und durch Vermehrung in ihr die Pellagra erzeugen würden. Auch Würmer, wie sie bei einigen Krankheiten bereits bekannt waren, konnte sich Gherardini ebenso wenig als Pellagra-Erreger vorstellen wie die Beschaffenheit der Luft. Also wendet er sich der Ernährung zu. Bei seinen Überlegungen kannte er offenbar nicht den bald nach Einführung des Anbaus von Mais in Europa erschienenen Hinweis von Giovanni Pietro Maffei [1536?–1603] aus dem Jahre 1600 (zit. n. [10]), dass Menschen bei ausschließlicher Maisernährung eine gewisse Schwäche zeigten.

Gherardini urteilte also, das Brot der armen Leute sei „ganz grob und aller guten Eigenschaften beraubt. Man findet es oft von nicht sehr dienlichem Teige von Mais oder Türkischem Korn mit Rocken oder Hirse gemacht, und was noch schlimmer ist, die meiste Zeit mit giftigem Unkraute vermischt, zum Beyspiele Trespe [*eine Grasart*], Lolch [*detto*], Wicken, blauen Kornblumen, Erven [*Wicklinse*], Hederich u.s.w., welche der schlechtesten Witterung ungeachtet in sehr großer Menge wachsen, und zum Besten gerathen, und wovon oft ein großer Theil mit auf die Kornböden der Landleute kommt. Die Wirkungen davon sind, dass eine verwickelte Reihe von Krankheiten daraus entsteht.“ Das gilt auch für schlecht gelagerte oder ungünstig zubereitete Speisen. Es „lässt sich ganz natürlich beweisen, dass die gewöhnlichen Speisen der Landleute die gelegentliche Ursache des Pellagra ausmachen; die davon erzeugte Schärfe [*der Körpersäfte*] die nächste, und der Sonnenbrand die entfernte Ursache desselben sei“. Es ist des Arztes Pflicht, „sich alle mögliche Mühe zu geben, durch Hilfe derselben einer sehr gefährlichen Krankheit vorzubeugen, wodurch die wichtigsten und notwendigsten Leute für den Staat [*die Bauern*] weggerafft werden“. Da das „vornehmste Nahrungsmittel zur Erhaltung der Landleute“ das Brot ist, soll gesorgt werden, „dass das zum Brotbacken dienliche Korn von allem giftigen Unkraute gereinigt, nicht eher, als wann es reif ist, eingeerntet, und hernach nicht zu sehr auf einander gehäuft würde“. Es ist auf das richtige Verhältnis von Wasser, Sauerteig und Mehl und auf die rechte Zeit für die Gärung zu achten. Bei Missernten des Korns [*Weizen*] kann man Gerste, Roggen oder Hafer verwenden.

„Wenn ungeachtet dieser Vorsicht das Pellagra wieder ausbricht, so müssen die Landphysici seine weitere Ausbreitung verhindern, dadurch dass sie zum Beispiel Molken mit irgend einem vorzüglich skorbutwidrigen Kraute zubereitet, oder, um noch ökonomischer zu sein, die bloße Abkochung von skorbutwidrigen Kräutern mit kühlenden und gelinde schweißtreibenden versetzt trinken lassen; die Gedärme von allen Unreinigkeiten befreien und fleißiges Baden oder Waschen empfehlen; denn durch bloßes wiederholtes Waschen und Reiben der Hände und Füße kann man es dahin bringen, dass sie nur die ersten Anfälle der Krankheit, und weiter nichts bekommen“.

Gherardini beschließt 1780 seine sehr umfängliche „Geschichte des Pellagra“ mit dem Hinweis, dass man hauptsächlich darauf zu sehen hat, zuerst allfällige begleitende Krankheiten „wegzuschaffen, um hernach die Heilung des Pellagra selbst freier unternehmen zu können“. Allerdings musste er bekennen, dass alle eigenen Versuche und auch die von ihm in der Literatur gefundenen, ihr Ziel, die Pellagra zu heilen, nicht erreichen konnten.

Weitergehende Gedanken über die Ursache der Pelagra äußerte 1778 Francesco Zanetti in einer Sitzung der Caesarea Leopldino-Carolina Academia Naturae Curiosorum [*„Deutsche Akademie der Naturforscher Leopoldina – Nationale Akademie der Wissenschaften“*]. Er schildert die Erfahrungen seiner sechsjährigen Betreuung von Patienten in der Insubria [11]. Dabei wandte er sein Hauptaugenmerk auf die Ernährung der dortigen Landbevölkerung.

Francesco Zanetti fand bei den von ihm betreuten Kranken die Ernährung als sehr bodenständig. Es gab Gerichte von Hülsenfrüchten oder von Hirse oder auch in gesalzenem Wasser oder in Öl gekochte Körner. Das Brot wurde aus Weizen- oder Gerstenmehl bereitet, aber vorzüglich aus den Feldfrüchten, welche die Italiener „Melega“ oder „Melica“ [*Mohrenhirse*], „Gran turco“ [*Türkenkorn*] und auch „Formentone“ nannten. Es ist dies der Mais, der oft schlecht gekocht und verdorben gegessen wurde. Als Getränke dienen seinen Patienten meist einfaches, an vielen Orten verunreinigtes Wasser oder auch Bier, zuweilen, aber selten ist es mit Wasser verdünnter saurer Wein aus Weintraubenkernen.

Die allgemeinen Ernährungsgewohnheiten der mitteleuropäischen Bevölkerung dieser Zeit beschreibt der Begründer der Volksgesundheitslehre, Johann Peter Frank (1745–1821), in seinem grundlegenden Werk „System einer vollständigen medicinischen Polizey“ [12], das ab 1779 in vielen Auflagen erschienen ist. Er schrieb nämlich: „Wir bedienen uns zu unserem Brode verschiedener Saamen grasartiger Gewächse: des Weizens, Rockens, der Gerste, Spelze des Hafers, so wie auch verschiedentlich des Buchweizens und des Welschkorns [*Mais*]. Unter diesen werden einige nur von ärmeren Menschen und in theuern Zeiten, bald allein, bald mit anderen Getreidegattungen vermischt, gebraucht.“

## Vorstellungen über die Pellagra-Genese

Als Ursache für das Auftreten der Pellagra galt ursprünglich die intensive Sonnenbestrahlung auf die Haut unbedeckter Körperteile.

In der Folge wurde aber dem Mais eine größere Bedeutung für die Pellagra zuerkannt. Dieser war keine bodenständige Feldfrucht, sondern ist etwa um 1530 durch spanische Seefahrer von den Antillen als neues „Getreide“ in ihre Heimat gebracht worden. Der Mais erwies sich bald als brauchbar für die Ernährung vieler Menschen. Sein Anbau in größerem Umfang begann in Spanien in der zweiten Hälfte des 16. Jahrhunderts und verbreitete sich von dort u. a. auch nach Frankreich und weiter nach Kleinasien und Ägypten. Aus der Türkei erreichte der Meis Italien um die Mitte des 17. Jahrhunderts. In die Donaufürstentümer [*Walachei und Moldau*] und damit an die Grenzen der habsburgischen Länder Siebenbürgen und Bukowina wurde der Mais erst kurz danach eingeführt [3]. Der langen Wanderung des „neuen Getreides“ entsprechend erhielt der aus dem indianischen Mahiz abgeleitete spanische Namen Maiz viele Synonyme wie Maïs, Mais, Kukuruz, Türkenweizen, Gran turco, Turkse tarwe, Welschkorn, (Indian) Corn und schließlich die botanische Bezeichnung Zea mays.

Der Vorteil der durch Mais erleichterten Ernährung besonders der armen Volksschichten war nicht ohne gesundheitlichen Nachteil, dem durch die spätere Einführung der Kartoffel als Volksnahrungsmittel begegnet wurde. In den Gebieten, wo der Mais das einzige oder fast ausschließliche Nahrungsmittel der armen Bevölkerung wurde, trat die Pellagra auf. Sie wurde im Laufe der Jahre in Spanien, Frankreich, Italien und Rumänien, aber auch in der Türkei, in Nordafrika und in Amerika festgestellt. Von Italien und Rumänien erreichte sie auch habsburgische Lande, im Südwesten die Lombardei, das Küstenland und Südtirol und im Osten die Bukowina.

Im Verbreitungsgebiet des Maises als hauptsächliches Nahrungsmittel gab es immer wieder Leugner seiner ätiologischen Bedeutung für die Pellagra. Die Königliche Akademie der Medizin in Madrid überreichte noch 1867 einen Spezialpreis an Juan Bautista Calmarza für ein Werk, in dem er in Übereinstimmung mit der Meinung fast aller iberischen Pellagrologen erklärte, dass die Ursache der Pellagra nicht dem Verzehren von Mais zugeschrieben werden kann [13].

Wenn man aber den Mais als „Schuldigen“ für die Pellagra ansah, so war dessen Wirkungsweise umstritten. Die als „Zeïsten“ [*von Zea mais*] bezeichneten Untersucher, wie insbesondere Gaetano Strambio sen. (1752–1831) und Giovanni Battista Marzari (1755–1827), waren der Meinung, der Mais verursache als ungenügendes Nahrungsmittel die Pellagra. Sie sei also eine Mangelkrankheit. Diese Vorstellung war sehr naheliegend, wenn man aus der mitteleuropäischen Wirtschaftsgeschichte ersieht, dass das 18. Jahrhundert von durch klimatische Krisen verursachte Hungerperioden geprägt war. Ab 1765 war die Witterung zunehmend feuchter geworden, was zu Missernten und in den Jahren 1770 und 1771 zur größten Hungersnot des Jahrhunderts geführt hat. Im Speziellen für den Mais brachte die Feuchtigkeit besonders schlechte Bedingungen der Konservierung.

Die Meinung, der Mais sei an sich zu nährstoffarm, wurde späterhin widerlegt durch den Beweis, dass der Eiweißbedarf der arbeitenden Landbewohner durch gewöhnliche Maiskost ganz gut gedeckt wird.

Eine andere Vorstellung von der ätiologischen Funktion des Mais war die Infektionsgenese. Diese hatte aber bereits Frapolli 1771 durch seine auf eigenen Beobachtungen beruhende Aussage „Interea neminem arbitror somniaturum Pelagram morbum esse contagiosum“ [*Jedoch solle niemand träumen, die Pellagra sei eine kontagiöse Krankheit*] abgelehnt [1]. Die Infektionsgenese wurde im letzten Viertel des 19. Jahrhunderts aber wieder interessant.

Unterdessen jedoch äußerte im Jahr 1818 Giammaria Zecchinelli (1776–1841) in Padua bei Betrachtung der Verhältnisse der Pellagra in den nordöstlichen Provinzen Italiens eine neue Meinung. Er schrieb, an der Erblichkeit der Pellagra zweifle jetzt niemand mehr; um sie erblich zu machen, müsse ein Elternteil in höchstem Grade pellagrinös sein; auf Neugeborene könne die Pellagra nur von einer Schwangeren oder Säugenden übertragen werden; in Familien, in denen das Übel erblich ist, sähe man nicht nur eine größere Anzahl pellagröser Kinder, sondern auch häufiger die versteckte Pellagra sowie die pellagröse Manie und garstige Geschwüre an Armen und Füßen; zwei- bis dreijährige Kinder würden zuweilen wahnsinnig [14]. Diesbezüglich ist auf eine noch 1944 erschienene Publikation von Bean, Spies und Blankenhorn zu weisen, die auf Grund ihrer ausgedehnten Literaturstudien und eigener Erfahrungen feststellten, dass Pellagra wohl kongenital [*recte: konnatal*], also angeboren sein kann, aber nicht hereditär [*erblich*] ist. Bei Neugeborenen und Säuglingen von an Pellagra leidenden Frauen kann nämlich diese Krankheit durch Mangel des Niacins in utero oder während der Laktation auftreten [15], wie wohl Niacin 1944 bereits als Pellagra-Präventiv-Faktor bekannt war.

## Ist die Pellagra doch eine Infektionskrankheit?

**Figure 4:**
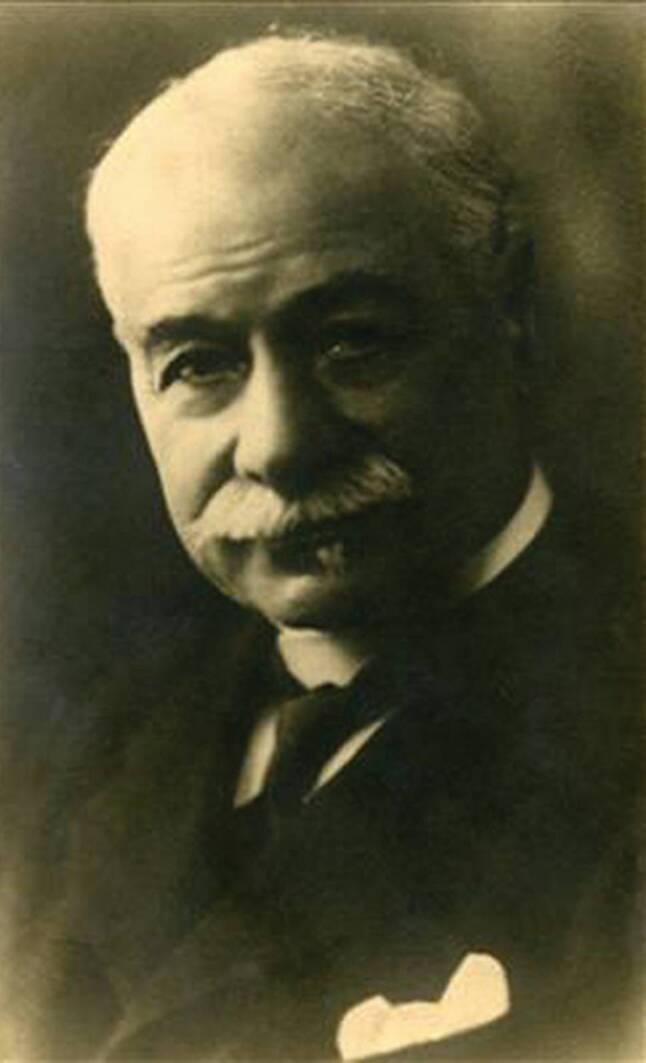

Mit dem Siegeszug der Bakteriologie im 19. Jahrhundert hatte man die Infektionsgenese wieder aufgegriffen. So berichtete Domenico Maiocchi (1849–1929) im Oktober 1881 in der Königlichen Medizinischen Akademie in Rom über seine vorläufigen Untersuchungen von Blut pellagröser Kranker und von aus verdorbenem Mais erzeugtem Mehl [16]. Im Blut zeigte sich „eine Myriade total beweglicher kleinster leicht ovaler Granuli, die isoliert, zu zweit oder in Ketten lagen oder moniliforme Filamente [*monile (lat.)* *=* *Perlenkette*] bildeten“. Wenn er das Blut 24 oder 48 h stehen ließ, war die Anzahl der Ketten und Filamente vermehrt. Im Mehl aus verdorbenem Mais konnte er gleiche Gebilde sehen. Soweit ihm bekannt war, fand man diese nicht im Blut Gesunder oder an anderen Krankheiten Leidender. Maiocchi hielt seine Befunde wichtig für die Ätiologie der Pellagra. Er nannte den gefundenen Keim Bacterium maydis. [*Der Name wurde später auf B. maïdis korrigiert (maïdis* *=* *Genitiv von mais).*] (Abb. 4).

**Figure 5:**
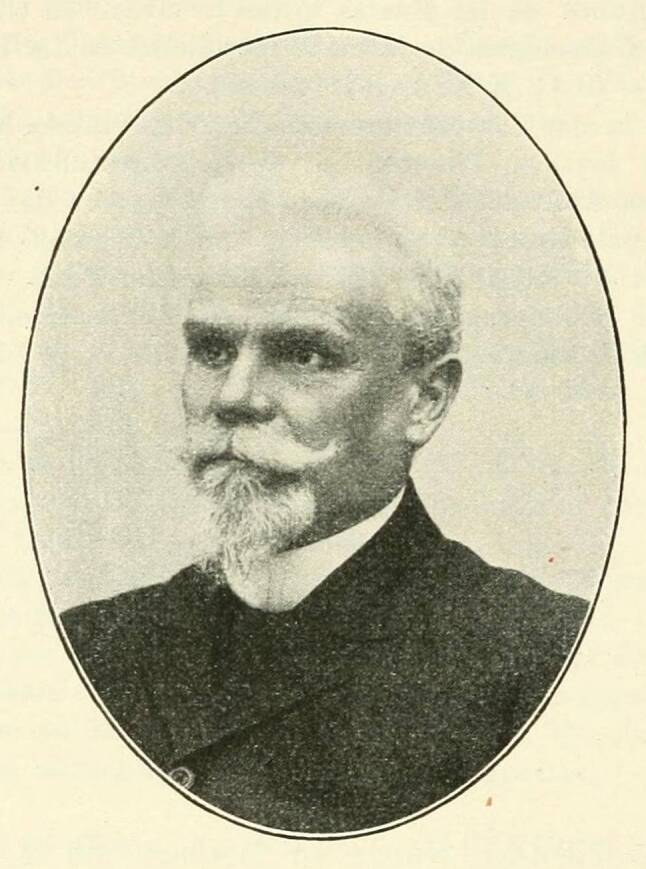

In den folgenden Jahren führte der Arzt und Botaniker Giuseppe Cuboni (1852–1920) während seiner Zeit als Professor in der Weinbauschule in Conegliano, Provinz Treviso, also einer Pellagra-Gegend, weitere bakteriologische Untersuchungen durch [18, 19]. Er fand ebenfalls ein vermeintlich gleiches Bakterium, jedoch nur in unreifem, in feuchter Umgebung gelagertem Mais (Abb. 5).

Cuboni stellte die Überlebensfähigkeit dieses Bakteriums bei 100 °C fest, wodurch es nach Genuss von aus solchem Mais erzeugter, also gekochter Polenta in den Fäzes der Bauern nachweisbar war. Auch in den Fäzes eines Hundes, der ausschließlich mit Mehl aus verdorbenem Mais gefüttert worden ist, fand Cuboni eine sehr starke Entwicklung des Bacterium maydis. Cuboni untersuchte auch das Blut von Pellagrösen in verschiedenen Stadien, um die Behauptungen von Maiocchi zu verifizieren, dass man in bestimmten Perioden dem Bakterium begegnet und daher die Pellagra zu den Infektionskrankheiten gehöre. Seine Blutkulturen waren aber immer negativ.

Durch die Untersuchung von 40 Pellagrapatienten kam Cuboni zur Hypothese, die Pellagra sei, wie die Cholera, eine durch übermäßige Entwicklung eines Bakteriums im Darm verursachte Krankheit. Hier also verursacht durch das Bacterium maydis, das mit der Polenta aus verdorbenem Mais vermehrungsfähig in den Darm gekommen ist.

Da die Pellagra als Volkskrankheit zunehmend gesteigertes politisches Interesse erlangte und die Genese noch immer nicht geklärt war, erweckte sie auch die Beachtung der Politiker und Behörden. So sah sich das k.k. Ministerium für Cultus und Unterricht in Wien im Frühjahr 1886 veranlasst, den Assistenten an der Wiener Internen Universitätsklinik, Dr. Edmund Neusser (1852–1941), zum Studium des „Wesens, ihrer Ursache und Prophylaxis“ der nun bedrohlich erscheinenden Krankheit ins österreichische Friaul und nach Rumänien zu senden [5, 6, 21].

Der Zeitpunkt der Entsendung Neussers war auch deswegen günstig gewählt, weil der Siegeszug der jungen Bakteriologie auch die Pellagra erreicht hatte. So wurden auch die klinischen und epidemiologischen Forschungen Neussers ergänzt durch bakteriologische Untersuchungen des Assistenten des pathologisch-anatomischen Instituts, Dr. Richard Paltauf [21, 22]. Nach seinen vorausgehenden gezielten bakteriologischen Studien in Italien stellten ihm die Barmherzigen Brüder in ihrem Spital in Görz einen Arbeitsplatz zur Verfügung.

Neusser kam auf Grund seiner Untersuchungen zum Schluss, die Pellagra sei das Ergebnis von Genuss verdorbener Polenta und krankhaftem Zustand der Polenta-Esser. Im verdorbenen Mais würden sich unter dem Einfluss des Bact. maïdis Vorstufen eines pellagragenen Giftes bilden, das bei Magendarmstörungen zu einem wahren Gift umgewandelt und nicht mehr ausgeschieden wird. Es wäre dies also eine gastrointestinale Autointoxikation. Die Abspaltung könnte unter Umständen auch bei der Schnapsdestillation erfolgen, wodurch der Schnaps Träger des Pellagragiftes würde. Nach Genuss solchen Schnapses entstünde die Pellagra als eine direkte Intoxikation.

**Figure 6:**
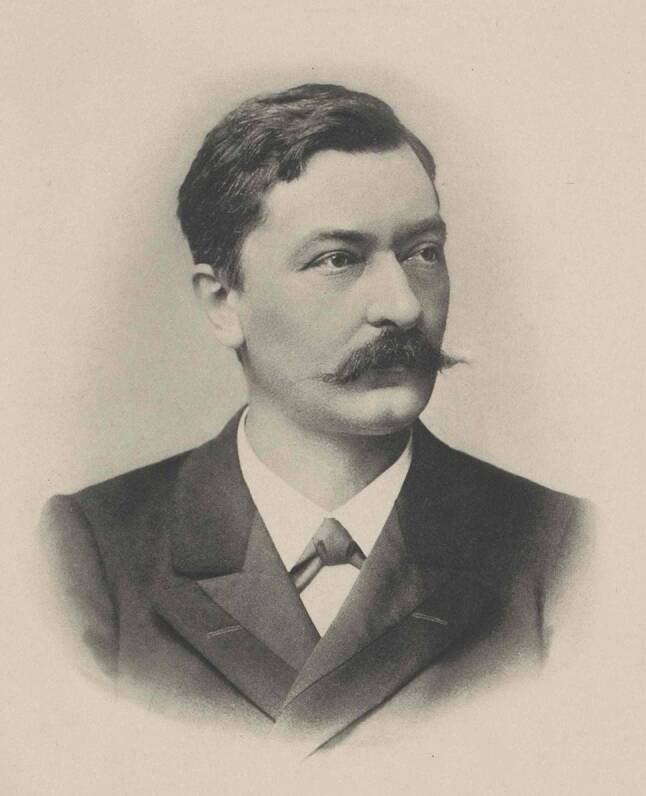

Diese Vermutung wurde in späteren Jahren (1905) – Neusser war unterdessen seit 1893 Vorstand der II. Medizinischen Klinik – von seinem Assistenten Adriano Sturli (1873–1964) bestätigt. In Rumänien hatte dieser nämlich beobachtet, dass viele Pellagröse, die eine Maisnahrung leugneten, Alkoholiker waren. Er kam also zum Schluss, dass die sogenannte „Pellagra ohne Mais“ vom reichlichen Konsum des üblicherweise aus verdorbenem, also billigem Mais erzeugten Branntweins stammen könnte [23, 24]. Dieser wird übrigens auch, mit Trebernbranntwein verschnitten, in andere Länder exportiert. Robert Flinker, Chefarzt des Psychiatrischen Krankenhauses in Czernowitz/Bukowina, zitiert eine Anzahl von Ärzten in der Bukowina und anderen Ortes, welche die gleichen Erfahrungen wie Sturli gemacht haben ([25]; Abb. 6 und 7).

**Figure 7:**
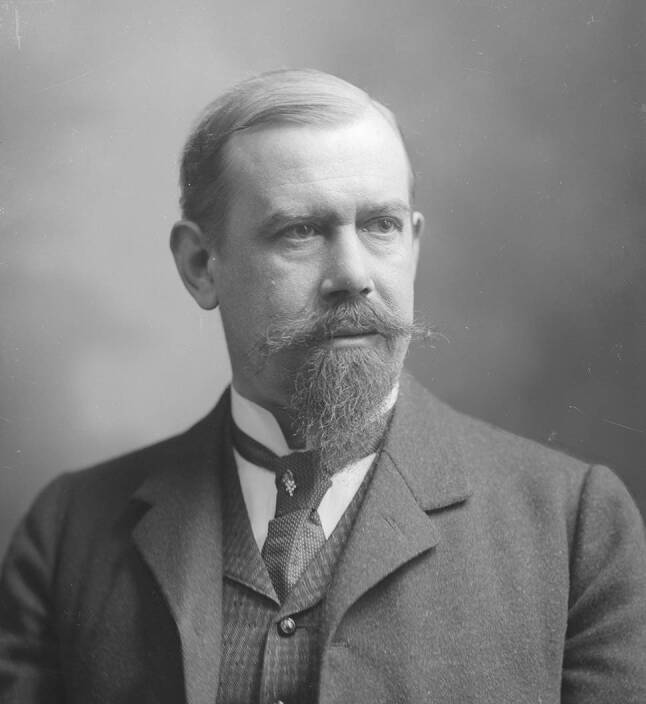

Rudolf Paltauf berichtete 1888 über seine in Wien durchgeführten bakteriologischen Kulturversuche an Maiskörnern [22]. Nachdem die gezüchteten Keime bei seinem Besuch bei Cuboni in Coneglio von diesem nach ihrer Form, Größe und dem kulturellen Verhalten als identisch mit dessen Bacterium maïdis bezeichnet worden waren, stellte Paltauf gemeinsam mit A. Heider in Wien umfängliche Untersuchungen an. Auf Grund der festgestellten Einwirkungen des Bakteriums auf Eiweißkörper, Kohlenhydrate, Milch, Ammoniak und Nitrate in vitro stimmten die Beiden Cubonis Meinung zu, dass das Bakterium im Maismehl giftige Stoffe bilden kann. Weiße Mäuse, die mit verschiedenen Zubereitungen von mit B. maïdis bewachsenen Nährmedien und Maismehlproben subkutan injiziert worden sind, zeigten verschiedene Reaktionen bis zu Lähmungen.

Aus ihren Untersuchungen kamen Paltauf und Heider zum Schluss, „dass die Pellagra keine mycotisch-parasitäre Krankheit ist, auch nicht im Sinne einer intestinalen Mycose Cuboni’s, dass nämlich der Bac. maïdis den Darm der Pellagrösen constant bewohne“. Sie halten dieses Bakterium für einen der weit verbreiteten Kartoffelbazillen, die „im Maismehl eine toxische, auf das Nervensystem weißer Mäuse narcotisch und lähmend einwirkende Substanz“ erzeugen.

[*Die von Paltauf gezüchteten Stämme von Bac. maïdis wurden in die Prager „Králsche Sammlung von Mikroorganismen“ aufgenommen, die nach Králs Tod in das Serotherapeutische Institut im Gebäude des Hygiene-Instituts der Universität Wien übersiedelt wurde (Franz Král, 1846–1911). Diese Sammlung, die sehr viele pathogene Mikroorganismen enthielt, musste 1945 auf Befehl der russischen Besatzungsmacht vernichtet werden.*] [26].

## Ist die Pellagra eine Vergiftung?

Nicht nur von Paltauf und Heider wurde gewissen an sich harmlosen Bakterien und Pilzen die Fähigkeit zugeschrieben, insbesondere im unreifen oder verdorbenen Mais Gifte bilden zu können, die von der sich mit solchem Mais ernährenden armen Bevölkerung aufgenommen werden.

**Figure 8:**
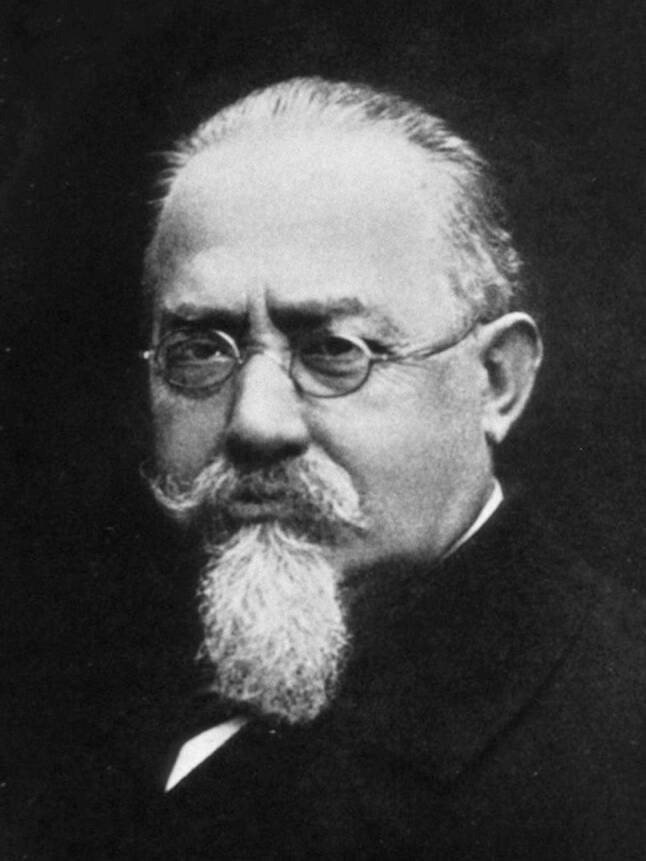

Immer wichtiger als die „Zeïsten“ wurde die Gruppe von Pellagraforschern, die eine Giftwirkung für die Auslösung der Pellagra annahmen. Diese „Toxikozeïsten“ mit Cesare Lombroso (1835–1909), Professor für Gerichtsmedizin, Hygiene und Toxikologie in Pavia, an der Spitze meinten, in unreifem Mais oder in zwar reifem jedoch nicht gut getrocknetem oder in schlecht gelagertem Mais oder in schlecht aufbewahrtem Maismehl und ungenügend gebackenem Maisbrot könnten durch harmlose Bakterien und Schimmelpilze giftige, pellagrogene Produkte gebildet werden. Tatsächlich isolierte Lombroso 1875 aus verdorbenem Mais verschiedene Stoffe, die in intaktem Mais fehlten [27]. Sie dürften aber für die Pellagra offenbar ohne Bedeutung sein wie Lombrosos Tier- und Selbstversuche gezeigt haben. Aber selbst aus völlig gesundem Mais kann man durch künstliche Fermentation Gifte mit großer Aktivität erlangen: Oleoresin, Pellagrozeïn, Maisharz (sostanza resinosa del mais), Maïsin. Der Toxikologe Theodor Husemann (1833–1901) bestätigte Lambrosos Befunde [28]. Er selbst machte mit solchen Stoffen Versuche an Fröschen, Salamandern und Kaninchen (Abb. 8).

Auf dem 5. Italienischen Pellagrakongress 1912 berichtete eine Gruppe von Forschern, dass Pellagrapatienten durch subkutane und intramuskuläre Injektionen eines wässrigen Extrakts aus verdorbenem Mais (Pellagrogenin) mit ausgesprochener Hypersensibilität (Schläfrigkeit, Stupor, halbkomatöse Zustände, starke nervöse und psychische Erregung, Temperaturen über 40 °C, blutige Durchfälle) reagierten, nicht aber bei Injektion von Extrakten aus gesundem Mais [30]. Diese Befunde stehen im Widerspruch zu den kurz davor von Raubitschek bei Pellagrakranken festgestellten [31, 32].

Einen anderen Aspekt allfälliger Giftwirkung bearbeiteten einige Untersucher. Sie fanden, dass über längere Zeit mit Mais gefütterte weiße Mäuse, Meerschweinchen und Kaninchen bei Haltung im Dunklen gesund blieben, bei Haltung im Licht entwickelten sich dagegen Hautveränderungen, Darm- und nervöse Symptome. Wie Letztere gehaltene, aber pigmentierte Tiere zeigten diese Symptome nicht oder nur sehr geringfügig [31–36]. Daraus wurde geschlossen, dass der „gesunde“ Mais einen sensibilisierenden Stoff enthält, der im Dunkeln ungiftig, im Licht aber giftig ist. Für solche photodynamisch wirkenden Substanzen gibt es verschiedene Kandidaten. Tatsächlich ist eine andere Feldfrucht, die auch der Ernährung des Menschen dient, der Buchweizen (Fagopyrum esculentum, Gran saraceno, Heiden) aus der Familie der Knöterichgewächse, für sensibilisierende Stoffe bekannt. Durch Verfütterung des Buchweizens an Rinder, Schweine und Schafe entstehen charakteristische Veränderungen der dem Licht ausgesetzten unpigmentierten Hautstellen und in der Folge kann der Tod eintreten. Die Pellagra könnte also eine der Buchweizenkrankheit (Fagopyrismus) analoge Erkrankung sein. Ausschließliche Maisfütterung der genannten Haustiere verursacht jedoch keine Erkrankungen.

Zu einem ähnlichen Schluss kommt Raubitschek [31, 32] bei seinen serologischen und Anaphylaxie-Untersuchungen an Patienten in „pellagrösen Gemeinden“. Er hält es für wahrscheinlich, dass aus den alkohollöslichen Anteilen des Kornes (Lipoiden), in den dem Sonnenlicht ausgesetzten Partien der Haut eine Noxe entsteht, die neben den lokalen Hauterscheinungen auch auf den Gesamtorganismus deletär wirkt.

Im Jahre 1905 kam eine neue Idee zur Infektionsgenese der Pellagra aus England. Auf der Tagung des Pellagra Investigation Committee am 21. Jänner 1905 stellte Louis W. Sambon (1865–1931) von der London School of Tropical Medicine seine neue Hypothese vor, die er 1910 ausführlich publizierte. Er vertrat nämlich die Meinung, die Pellagra werde durch ein unbekanntes Protozoon verursacht, das Simulien durch ihren Stich beim Blutsaugen übertragen [13]. Simulien (Kriebelmücken, Sandfliegen) sind 3–6 mm lange, fliegenähnliche Mücken, deren Larven nur in stark fließendem Wasser leben. Sambon begründet seine Behauptung damit, dass die Krankheit nur in ländlichen Distrikten (nicht in Städten) vorkommt und die Imagines der Simulien saisonal wie die Pellagra im Frühjahr und Herbst auftreten und dass für einige Jahre Rückfälle im Frühjahr auch dann auftreten, wenn der Patient aus der Pellagraregion entfernt wurde. Letzteres zeigt, dass die Krankheit nicht direkt kontagiös ist. Er sagt, dass Pellagra auch dort auftritt, wo Mais nicht konsumiert wird, aber dass es wo immer Pellagra auftritt Simulien gibt [*was nicht wundert, da diese weit verbreitet sind*]. Sambons Hypothese fand in den USA, wo es zum Zeitpunkt der Publikation (1910) eine Zunahme der Pellagra in 22 Bundesstaaten gab [37], einen Unterstützer [38]. Bei europäischen Untersuchern stieß sie aber wegen ihrer falschen Prämissen auf sehr verständliche Ablehnung [10, 34, 39, 40]. Diese wurde vom Landessanitäts- und Pellagrainspektor E. Weiß in Innsbruck in der praktischen Anschauung dargestellt [40]. Er hatte nämlich im September 1911 den dienstlichen Auftrag erhalten, Sambon mit einem englischen Pathologen auf einer Reise durch das italienischsprachliche Südtirol zur Untersuchung des Verhältnisses von Pellagra und Simulien zu begleiten.

Die vorgeschrittene Jahreszeit der Reise war „für das Auffinden akuter oder deutlicher Pellagrafälle nicht günstig“. Es wurden Pellagrazentren und Gegenden, wo die Pellagra angeblich nicht vorkommt (Cavalese, Tesinotal/Bezirk Borgo) bereist. Die dabei von Sambon getätigten Erklärungen und Interpretationen veranlassten Weiß und den Innsbrucker Professors Ludwig Merk (1862–1925) zur späteren Feststellung, sie hätten „das Empfinden, als solle einer Schreibtischhypothese eine Reihe von Tatsachen angepasst werden“.

Dem Zeitgeist entsprechend wurde auch diskutiert, ob die Pellagra eine virale Infektion ist. Diesbezügliche Tierversuche an drei Rhesusaffen führte William H. Harris 1913 in den USA durch [41]. Er injizierte subkutan, intravenös und intrakranial Berkefeld-Filtrate von Suspensionen von Teilen des Gehirns und Rückenmarks, von Hautläsionen, des Darmtrakts und der Nasen-Rachen-Mukosa von an Pellagra Verstorbenen. Harris kommt zu dem Schluss, „die Experimente zeigten, dass die Pellagra auf Rhesusaffen durch Berkefeld-Filtrate menschlichen Gewebes übertragen werden kann; zumindest entwickelten die Tiere die wesentlichen klinischen Zeichen und Symptome zusammen mit dem pathologischen Bild bei der Erkrankung des Menschen“. Weitere virologische Untersuchungen sind mir nicht bekannt.

## Klärung der Pellagra-Ursache

Die Suche nach der Ursache der Pellagra bekam einen neuen Impuls, nachdem um 1900 in den USA die Bauern begonnen hatten, anstelle von Weizen und Gerste wegen des höheren Ertrages Mais anzubauen. 1906 begann diese Krankheit als Seuche die östlichen Südstaaten der USA heimzusuchen. Bis 1940 wurden ca. 3.000.000 Fälle registriert, von denen 100.000 starben. So wurden im Jahre 1912 allein im Staate South Carolina 30.000 Fälle mit einer Letalität von 40 % gemeldet. Der US-Kongress verlangte daraufhin eine genaue Untersuchung, die 1914 dem beim U.S. Public Health Service arbeitenden Joseph Goldberger (1874–1929) übertragen wurde. Dieser hatte schon Erfahrungen mit der Bekämpfung von Typhus, Gelb‑, Fleck- und Denguefieber in den USA, in Mexiko und in der Karibik [42–49].

Auf Grund seiner Feststellung, dass in Waisenheimen und psychiatrischen Spitälern die Verpflegten Pellagra entwickelten, während die Pflegenden gesund blieben, hielt er die unterschiedliche Ernährung für die Krankheitsursache. Seine Hypothese wurde bestätigt. Er veranlasste die Abgabe von normaler Kost mit Fleisch, Milch und Gemüse anstelle der auf Mais beruhenden Ernährung an Bewohner zweier Waisenhäuser und eines Altenheims mit dem Erfolg, dass die Pellagrakranken gesundeten und Gesunde nicht erkrankten. An einem Versuch im Jahre 1915 in einem Gefängnis nahmen elf Insassen gegen das Versprechen der nachfolgenden Freilassung teil. Sie erhielten nur auf Maisbasis beruhende Speisen. Nach fünf Monaten zeigten sechs Versuchspersonen Pellagrasymptome der Haut und klagten über fürchterliche Beschwerden.

Goldbergers Hypothese wurde von der Kollegenschaft in den USA, die allgemein an die Infektionsgenese glaubte, heftig widersprochen. Zum endgültigen Beweis injizierten am 26. April 1916 Goldberger und sein Assistent George Alexander Wheeler (1885–1981) einander fünf bzw. sechs Milliliter defibriniertes Blut eines Pellagrösen in den Arm. Weiters spülten sie das Sekret aus Nase und Rachen eines Pellagrapatienten und rieben dieses in ihre Nasen und Rachen. In einer anderen Versuchsreihe schluckten sie Kapseln mit Geschabsel von pellagrösen Hautstellen. Die Versuche wurden von anderen Kollegen wiederholt. Alle Versuchspersonen blieben gesund. Trotzdem verblieben unter den Ärzten einige Opponenten von Goldbergers Diättheorie der Pellagra.

So startete Goldberger mit seinen Assistenten George Alexander Wheeler (1885–1981) und Virgil Preston Sydenstricker (1889–1964) die von ihm „Dreckparty“ genannten Selbstversuche. Es wurden Harn und Fäzes von Pellagrakranken mit etwas Weizenmehl zu kleinen Kugeln geformt und diese auf leeren Magen geschluckt. Goldbergers Frau bekam nicht solche Kugeln, jedoch wurde ihr Blut einer sterbenden Pellagrapatientin abdominal injiziert. Sieben solche „Dreckpartys“ wurden mit 16 Kollegen abgehalten. Alle Versuchspersonen blieben gesund.

1915 publizierten Goldberger und zwei Mitarbeiter (C. H. Waring und D. G. Willets), dass die Pellagra durch geeignete Diät (Milch, Eier, Fleisch, Bohnen, Erbsen) verhütet und auch geheilt werden könnte. Sieben Jahre später meinten er und W. F. Tanner, dass der „Pellagra-Präventiv-Faktor“ eine Aminosäure sein könnte [50]. Goldberger begann mit seinen Mitarbeitern experimentelle Studien mit Hunden, die an der Schwarzzungenkrankheit litten, einer der Pellagra analogen Krankheit. Diese konnte durch Leberextrakt bekämpft werden. Goldberger erlebte nicht mehr die Mitteilung des Biochemikers Conrad Arnold Elvehjem (1901–1962) von der Universität von Wisconsin im Jahr 1937 [50–52]. Dieser berichtete mit seinen Mitarbeitern R. J. Madden, F. M. Strong und D. W. Wooley, dass mit Nikotinsäure die Schwarzzungenkrankheit der Hunde geheilt werden kann [53]. Es gelang Elvehjem auch die Reindarstellung dieser Substanz, auch Pellagra-Präventiv-Faktor genannt, die er der inhomogenen Vitamin-B-Gruppe zuordnete. Spätere Studien von Tom M. Spies, Clark Cooper und Marion Blankenhorn mit einem Gemisch aus Nikotinsäure und Nikotinsäureamid, für das man zur Vermeidung des Hinweises auf das toxische Nikotin den Namen „Niacin“ geschaffen hat, bewiesen die gute Wirkung auch auf die Pellagra [54].

Durch Elvehjems Feststellung der ätiologischen Bedeutung des Niacins kommen wir nochmals an den Anfang des Maisimports durch die Spanier nach Europa [55]. Die indigenen Völker Mittelamerikas veränderten den von ihnen gezüchteten Mais vor dem Genuss. Die Aufbereitungsverfahren bestanden im Prinzip aus der Hitzeeinwirkung alkalischer Lösungen auf das Maiskorn. Solche Lösungen wurden aus Kalksteinen oder pflanzlichen Aschen hergestellt. Durch das Kochen wird die Kleie vom Kern abgelöst, das Eiweiß wird besser resorbierbar und das an Hemizellulose gebundene Niacin wird frei für die Aufnahme in den Körper. Außerdem wird der Gehalt an Mykotoxinen, zumindest von Fusarium-Pilzen, zu 90–94 % reduziert. Der Name dieses Verfahrens „Nixtamalisation“ wurde aus der Sprache der Azteken für Asche (nextli) und Mais-Teig (tamali) abgeleitet.

Schließlich sei noch die sekundäre Pellagra erwähnt. Von dieser spricht man, wenn bei einem Patienten, dessen Diät die notwendigen Bedürfnisse abdeckt, bei weiterhin gleicher Diät durch äußere Einflüsse die Pellagra auftritt und diese bei Korrektur der störenden Einflüsse wieder verschwindet [15].

## Pellagra in den k.k. Provinzen Küstenland und Tirol

**Figure 9:**
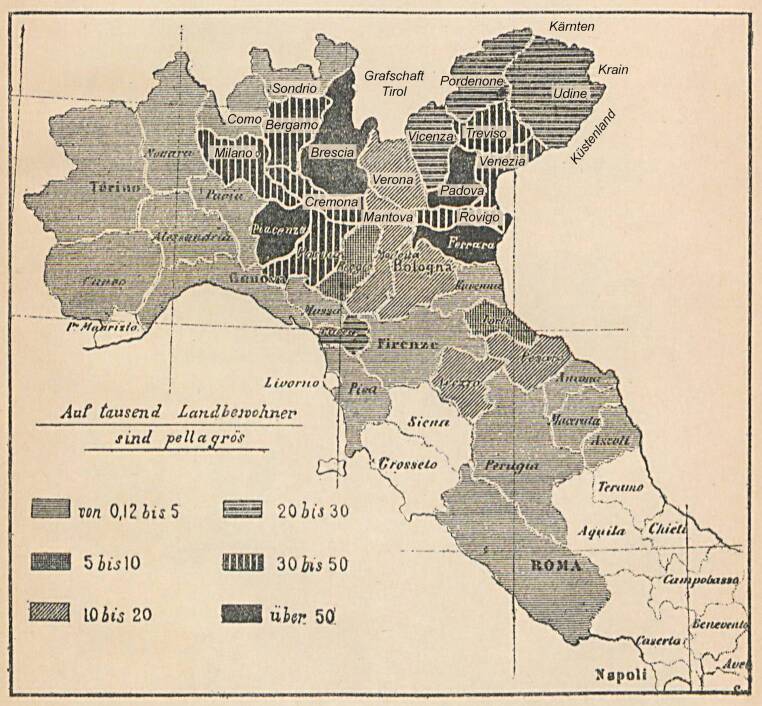

Die Pellagra herrschte im 18. Jahrhundert in den nördlichen Staaten Italiens sicher so verbreitet, wie es Angaben aus dem ab 1797 in Teilen vereinigten Königreich Italien vermuten lassen. Es werden da Häufigkeiten von 5 % und mehr in der ländlichen Bevölkerung angegeben (Abb. 9). Der erste Bericht war wohl Frapollis Beschreibung von 1771 aus der bis zum Frieden von Campoformio (1797) und wieder von 1815 bis 1859 österreichischen Lombardei. Die oben besprochenen Forscher stammten alle aus der Lombardei.

Das Endemiegebiet Norditaliens erstreckte sich jenseits der Grenze im südwestlichen Österreich hauptsächlich in flachen Regionen der k.k. Provinz „Küstenland“. Diese bestand aus der gefürsteten Grafschaft Görz und Gradisca, der Markgrafschaft Istrien und der reichsunmittelbaren Stadt Triest mit ihrem Gebiete und wurde 1866 um den nicht an das Königreich Italien abgetretenen Teil Friauls erweitert. Im Küstenland wurde die Pellagra zum ersten Mal im Jahre 1832 in den Büchern des Spitals der Barmherzigen Brüder in Görz erwähnt [57].

Als sich ab dem Jahre 1884 die Häufigkeit der Pellagra als Volkskrankheit gesteigert hatte, behandelte auch der Reichsrat die Frage der Pellagra in Österreich. In der 37. Sitzung der X. Session des Hauses der Abgeordneten am 27. März 1886 erbat der Abgeordnete von Görz, Graf Franz von Coronini, das Wort [58]. Er nahm den Budgettitel „Auslagen für Epidemie und Epizootie“ zum Anlass, auf die schwere Notlage eines Teils seiner Heimat hinzuweisen, „welche einst an die üppigsten Gestade Italiens erinnerte“. Es betraf dies insbesondere den politischen Bezirk Gradisca der gefürsteten Grafschaft. Durch verschiedene Ursachen, aber nicht zuletzt durch die „Regulierung der Grund- und Gebäudesteuer, welche in einem höchst ungünstigen Momente die Lasten der Steuerträger in bedeutendem Maße erhöht hat“, ist ein Teil der Bevölkerung in eine empfindliche Notlage geraten. Die Einen treibt die Not nach Amerika, die Anderen verfallen in eine Krankheit, in die Pellagra, „und diejenigen, welche wiederholt von dieser Krankheit befallen werden, endigen meist in der Nacht des Wahnsinns, in den Irrenhäusern“. Man sei im „benachbarten Italien daraufgekommen, dass eine wesentliche Besserung schon dadurch herbeigeführt werden könne, wenn man den Leuten ein besseres, gesundes und kräftiges Brot zu billigen Preisen liefern kann“. Die Ursache sei die ausschließliche Ernährung mit Mais, der in den Bauernhäusern auch oft nicht richtig konserviert wird. Im benachbarten Königreiche Italien gibt es seit 1884 die staatlich unterstützten „Forni rurali“ [*ländliche Backöfen*], „wo den Leuten ein gutes, billiges, nahrhaftes Brot geliefert wird. Es wäre also von höchstem Interesse, wenn in unseren Provinzen solche Institute auch errichtet würden“. Der Abgeordnete, Graf Coronini, bittet das hohe Haus um Annahme seiner Resolution: „Die k.k. Regierung wird aufgefordert, dem Auftreten der Pellagra volle Aufmerksamkeit zuzuwenden, zur Bekämpfung dieser Krankheit alle als geeignet befundenen Maßnahmen zu ergreifen und zu diesem Behufe auch die nothwendigen Geldmittel von der Volksvertretung in Anspruch zu nehmen“. Der Resolutionsantrag wurde, allerdings nicht einstimmig, aber immerhin „genügend unterstützt und wird der geschäftsordnungsmäßigen Behandlung unterzogen werden“.

Ein Jahr später wurde von anderen Abgeordneten wieder ein Antrag in diesem Sinne gestellt [59]: „Die k.k. Regierung wird aufgefordert, dem Auftreten der ,Pellagra‘ volle Aufmerksamkeit zuzuwenden und zur Bekämpfung dieser Krankheit alle als geeignet befundenen Maßregeln zu ergreifen.“

Schließlich wurde die Pellagra nach einem weiteren Jahr in der 198. Sitzung am 6. März 1888 behandelt [60]. In dem schriftlichen Bericht des Budgetausschusses (Beilage 540 zu den stenographischen Protokollen [61]) wird auf die im Jahre 1886 von der k.k. Regierung veranlasste Entsendung von Dr. Edmund Neusser in das österreichische Friaul aufmerksam gemacht, und die damals bekannten Fakten der Pellagra werden dargestellt. Es wird berichtet, dass seit dem Jahr 1884 die Pellagra in der Grafschaft Görz und Gradisca vermehrt auftritt. In den am meisten betroffenen Ortschaften erkranken 5–6 % der Bevölkerung, aber durchschnittlich 25 % „tragen die Pellagra mit sich“; 90 % der Pellagrabehafteten sind Taglöhner. Tatsächlich sind die angegebenen Zahlen zu niedrig, weil die Gemeinden die meisten, insbesondere die leichten Erkrankungsfälle aus Furcht vor kostspieligen, auf ihre Kosten vorzunehmende Maßnahmen nicht melden. Auch die offiziellen Sterbezahlen sind wegen verschiedener angegebener Todesursachen nicht brauchbar.

In dieser Sitzung wurde auch das „Gesetz vom 31. März 1888, betreffend die Gewährung von Unterstützungen aus Staatsmitteln zur Bekämpfung der Pellagra-Krankheit und zur Linderung des Nothstandes in der gefürsteten Grafschaft Görz und Gradisca“ beschlossen [60]. Darin wird zur Unterstützung der durch die Missernte des Jahres 1887 notleidenden Bevölkerung eine Gesamtsumme von 50.000 fl. [*Florin* *=* *Gulden*] bewilligt. Daraus sind 20.000 fl. „zur Bestreitung des Aufwandes zu verwenden, welcher sich aus der Anwendung der zur Bekämpfung der Pellagra-Krankheit erforderlichen besonderen prophylaktischen und sanitätspolizeilichen Maßregeln, wie beispielsweise der Einrichtung von Back- und Trockenöfen, der Beschaffung gesunder und zweckmäßiger Nahrungsmittel, sowie von Heilmitteln für die von der Krankheit ergriffenen, der Assanierung von gesundheitsschädlichen Häusern, welche von Pellagra-Kranken bewohnt werden u.d.gl. ergeben wird“. Aus der Gesamtsumme kann ein Betrag von 30.000 fl. „zur Ausführung von öffentlichen gemeinnützigen Bauten und unter besonders rücksichtswürdigen Umständen zur Beschaffung von Lebensmitteln“ verwendet werden.

Weiters wurden in der Sitzung noch zwei Resolutionen angenommen. Die eine fordert von der Regierung, die zur Bekämpfung der Pellagra nötigen Mittel auch für die kommenden Jahre zu budgetieren. In der anderen Resolution wird die Regierung aufgefordert, „über das Vorkommen der Pellagra und das Gebiet ihrer Ausdehnung in Südtirol die entsprechenden Erhebungen zu pflegen und eventuell die nöthigen Vorkehrungen zu treffen“.

Als Häufigkeit der Pellagra im österreichischen Friaul fand Neusser unter den 36.588 Bewohnern 1068 Pellagröse (2,92 %), von denen 96 (9 % bzw. 0,26 % der Gesamtbevölkerung) „Irrsinnsfälle“ waren [5, 21].

Im April 1888 wurde erstmals über die Auswertung der gemäß Erlass der küstenländischen Statthalterei vom 20. April 1884 [57] von den Ärzten verpflichtend ausgefüllten Zählblätter berichtet. Aus 28 der 70 Gemeinden der Bezirkshauptmannschaft Gradisca wurden Pellagrafälle gemeldet, die zwischen 0,05 und 6,75 % der Ortseinwohner betrafen. „Die Pellagra hat ihren Sitz in den tief gelegenen Theilen von Friaul, den sogenannten Basse, kommt in höher gelegenen Gegenden nur sporadisch vor“, weil die Bewohner der gebirgigen Bereiche kaum Mais konsumieren.

In den italienischsprachigen Gegenden Südtirols kennt man die Pellagra zumindest seit den Jahren 1791 und 1792, in deren sie in den Totenbüchern der Gemeinden Pomarolo und Vallarsa des Bezirks Rovereto [*Rovereid*] vermerkt ist. Im 19. Jahrhundert scheint sie in den der lombardo-venezianischen Grenze benachbarten Gemeinden ziemlich verbreitet gewesen zu sein. Die erste Erhebung (Frühjahr 1888) ergab in 115 der 364 Gemeinden 633 gemeldete Pellagrafälle [57]. Ihre lokale Häufigkeit lag bei jeweils einem Fall auf 47 bis auf 1980 Einwohner, wobei die Städte Trient und Rovereto mit nur zwei bzw. vier gemeldeten Fällen außerhalb dieser Berechnung lagen. In den folgenden Jahren nahmen in Südtirol die Zahlen der Pellagakranken und der betroffenen Gemeinden sukzessive ab, bis sie aber im Jahre 1895 wieder anstiegen [62].

Gesamtangaben über die Häufigkeit pellagröser Geisteskrankheiten fehlen. Die Statistik der Landesirrenanstalt zu Pergine [*Pergen*] vom 1. Jänner 1889 bis 10. November 1894 [57] führte unter der Gesamtzahl der verpflegten Geisteskranken 19,5 % mit Pellagra Behaftete. Unter den 190 Entlassungen von Pellagrösen waren 25 Todesfälle.

Über die Sterbefälle in Folge von Pellagra liegen keine vollständigen Nachweisungen vor. Im politischen Bezirk Rovereto lagen die jährlichen Zahlen in den Jahren 1889–1895 zwischen 13 und 33, insgesamt waren es 164 Fälle [57].

Beim Vergleich von Südtirol und dem Bezirk Gradisca fällt ein wesentlicher Unterschied auf. In Südtirol wird die Krankheit in einer ungleich größeren Zahl von im ganzen Landesteil zerstreuten Gemeinden beobachtet, wobei aber in der weit überwiegenden Mehrzahl der Gemeinden nur vereinzelte Fälle verzeichnet sind, während im politischen Bezirk Gradisca ein eng umgrenztes Endemiegebiet mit einer weit größeren Zahl von Pellagrösen vorliegt.

## Pellagra in der Bukowina (und in Galizien?)

Wiewohl es bekannt war, dass die Pellagra in den Fürstentümern Moldau [*östlich der Bukowina*] und Walachei [*südlich von Siebenbürgen*] und im 1862 durch deren Vereinigung entstandenen Rumänien beobachtet wurde, wunderte es, dass keine Meldungen aus dem angrenzenden, seit 1774 österreichischen Kronland Herzogtum Bukowina [*Buchenland*] mit der Hauptstadt Czernowitz bekannt waren. Dies erschien dem Czernowitzer Krankenhausarzt Philipowicz 1888 umso befremdlicher, als die Lebensweise der Bukowinaer Landbevölkerung jener der rumänischen durchaus ähnlich war. Seine Skepsis wurde durch die Tatsache gestärkt, dass das Klima in Rumänien die Reifung des Maises begünstigt, während in der Bukowina durch das strengere Klima mit frühzeitigen Frösten der zu dieser Zeit noch nicht reife Mais geschädigt oder an der Ausreifung gehindert wird. Fast alle Pellagrösen gehörten zu den allerärmsten Landbewohnern, welche in den elendsten Verhältnissen lebten und weder Milchtiere, noch gar Grund besaßen. Das galt fast ausnahmslos für die bodenständige rumänische oder ukrainische Bevölkerung der Bukowina und nur ausnahmsweise für die seit drei Generationen dort ansässigen bäuerlichen Kolonisten (Deutsche, Ungarn, Polen und Lippowaner) und die zahlreichen Israeliten [25, 63, 64]. [*Lippowaner: Nachkommen der im 18. Jahrhundert aus dem Zarenreich in die Donausümpfe Bessarabiens geflohenen russischen Leibeigenen altorthodoxen Glaubens.*]

Philipowicz, der in Görz die Pellagra kennen gelernt hatte, konnte tatsächlich in der Allgemeinen Landeskrankenanstalt in Czernowitz im Jahre 1887 sechs Pellagra-Erkrankungen diagnostizieren und drei daran Gestorbene obduzieren.

Der Bezirksarzt in der Stadt Suczawa an der moldauischen Grenze und spätere Bukowinaer Landesregierungsrat Basil Kluczenko beobachtete 1889 zwölf an Pellagra Erkrankte und zwei weitere, die wegen dieser Erkrankung schließlich Selbstmord begangen haben [64]. Er berichtete auch, dass der einheimischen Bevölkerung seit Dezennien die Krankheit bekannt sei und diese in früheren Jahren, insbesondere nach Missernten häufiger aufgetreten sei als in neuerer Zeit. Die Bewohner der Gebirgsgegenden seien verschont geblieben. Seiner Meinung nach könnte auch in den nördlich an die Bukowina angrenzenden Bezirken des österreichischen Kronlandes Galizien, in denen sich die Landbevölkerung ebenfalls vorwiegend mit Mais ernährt, höchstwahrscheinlich auch Pellagra vorkommen. Kutakowski aus Tarient in Galizien berichtete jedoch, dass bei den Dorfbewohnern Galiziens an der Grenze zur Bukowina Pellagra nicht bekannt ist [65]. Sie trocknen den Mais vor dem Mahlen und mahlen nur für jeweils vier Wochen, damit das Mehl nicht zu lange lagert und dabei vergärt.

Erst 1903 erwähnt Würzel, Primarius am A. ö. Krankenhaus in Suczawa, er habe bereits 1883 wahrscheinlich den ersten Pellagrafall in der Bukowina diagnostiziert [66].

Die im Bezirk Suczawa und in der Landeskrankenanstalt in Czernowitz festgestellten Fälle veranlassten die Bukowinaer k.k. Landesregierung, an alle Bezirkshauptmannschaften den „Erlass vom 19. Mai 1891, Z. 6998, betreffend Erhebungen über das Vorkommen der Pellagra,“ zu richten [67]. Die Bezirksärzte und andere mit der Pockenimpfung im Sommer betrauten Ärzte wurden beauftragt, „der Ausbreitung der Pellagra gelegentlich der Vornahme der Sommer[*pocken*]impfung in den einzelnen Gemeinden nachzuforschen, die vorgefundenen Pellagrösen zu untersuchen und den Befund“ in ein vorgegebenes Zählblatt aufzunehmen. Die Sommerzeit wurde gewählt, weil durch die erhöhte Sonnenbestrahlung die pellagrösen Hautveränderungen deutlicher sind. Die Anzahl amtlich festgestellter Pellagrafälle stieg von acht im Jahr 1887 im Folgejahr auf 152 und im Jahr 1900 auf 227. Im Bezirk Suczawa betrug der Anstieg zwischen 1887 und 1903 von zwei auf über 400 Fälle im Jahr [34]. Auch weiterhin nahmen die Erkrankungszahlen in den folgenden Jahren zu [68]. Bei der Bevölkerung der Bergdörfer kam die Pellagra nicht vor [69]. Auch noch im Jahre 1910 wurde in einer Sitzung des k.k. Landtages die Pellagra als eine der gefährlichsten Volkskrankheiten der Bukowina bezeichnet [70].

Die Zunahme der Pellagramorbidität in der Bukowina steht „unzweifelhaft mit dem wirtschaftlichen Niedergange und der Verschuldung der bäuerlichen Bevölkerung in Zusammenhang“. Bei der steigenden Armut musste diese „in der Auswahl des zum Genuss bestimmten Maismehls (ihres Hauptnahrungsmittels) immer weniger wählerisch werden und verwendeten schließlich auch ganz verdorbenes, verschimmeltes Maismehl“ zur Herstellung ihrer Mamaliga, wie die Polenta in der Bukowina heißt. Dazu kam noch, dass die sich im Sommer wegen höherer Gehälter als landwirtschaftliche Arbeiter nach Rumänien verdingenden Bukowinaer von manchen gewissenlosen Pächtern und Gutsbesitzern zur Ernährung die schlechtesten Maissorten erhielten.

Die neuen Bahnanschlüsse nach Ungarn, Rumänien und Russland ermöglichten die Einfuhr großer Mengen von Mais, wodurch dessen Preis sehr gefallen ist und die Produktion in der Bukowina nicht mehr kostendeckend war. Außerdem wurde der lokale Maisanbau durch den Anbau von Kartoffeln und Rüben eingeschränkt. Als Folge all dessen wurde viel Mais aus dem Osten eingeführt, der sehr oft feucht ankam und von den Importeuren dann nicht richtig gelagert wurde. Das Verderben von Mais und des daraus bereiteten Mehls waren die Folge. Diese veränderten Verhältnisse wurden für die rasche Ausbreitung der Pellagra in der Bukowina anfangs des 20. Jahrhunderts verantwortlich gemacht [66, 69]. Es war auffällig, dass ein großer Teil der Landbevölkerung auf Maiskonsum beharrte und die Einführung der „Weißfrüchte“ (Roggen, Weizen) ablehnte. Da die Bukowinaer Bauern nicht wussten, was sie mit dem Getreide anfangen sollten, lehnten sie auch dessen Einbringung ab. Dafür mussten Arbeiter aus Galizien geholt werden, deren Lohn in Getreide abgegolten wurde [69].

Darüber hinaus wurde „mit Bestimmtheit ausgesprochen, dass die schlechte Lebensweise, das viele strenge Fasten und der übermäßige Genuss von Alkohol die Widerstandsfähigkeit der ländlichen Bevölkerung gegen die Pellagra herabsetzten und ihre Disposition für die Pellagra steigerten.“ Eine bessere Ernährung, eine Milderung oder Abkürzung des Fastens und eine Bekämpfung des Alkoholgenusses wurden demnach als notwendige Vorbedingungen einer Bekämpfung der Pellagra angesehen [69].

## Pellagra in Ungarn und in Rumänien

Die Berichte über die Pellagra in der anderen Reichshälfte Österreich-Ungarns, im Königreich Ungarn, sind vorwiegend in ungarischer Sprache erschienen, sodass ich mich vorwiegend auf die Veröffentlichung des in Wien promovierten Ungarn S. H. Scheiber (1834–1906) verlasse [70]. Dieser sehr aktive Chirurg, Prosektor und Anthropologe arbeitete zuerst in Wien, dann als Primarius und Universitätsprofessor in Rumänien in Iaşi [*Jassy*] und Bukarest, wo er Erfahrungen über die Pellagra sammelte, und zuletzt als Arzt in Ungarn in Székesfehérvár [*Stuhlweißenburg*] und Budapest.

In Ungarn wurde erstmals 1888 über die Pellagra berichtet, als die sog. Csángó-Ungarn [*röm.-kath. Ungarn aus der Region Moldau*], die sich mit Maismehl ernähren, aus der Bukowina nach Ungarn übersiedelt sind. In den folgenden Jahren wurde die Pellagra in einigen Siebenbürger und angrenzenden ungarischen Komitaten festgestellt. Im Jahr 1899 trat eine Pellagra-Epidemie im Klausenburger Komitat in Siebenbürgen auf. Neben den in die interne Klinik (Thomas Marschalko von Csépánfalva, 1862–1915) und in die Hautklinik (Zsigmond Purjesz, 1846–1918) der Universität in Klausenburg [*ungar.: Kolozsvár, heute: Cluj-Napoca, Rumänien*] aufgenommenen Pellagrapatienten wird ein an die Klausenburger Psychiatrische Klinik (Karl Lechner, 1850–1922) überwiesener pellagröser Geisteskranker erwähnt. [*Siebenbürgen war 1699–1848 und 1849–1867 ein mit Österreich verbundenes eigenes Großfürstentum (Verwaltung in Wien und Hermannstadt, rumän.: Sibiu), 1848–1849 und ab 1867 Teil des Königreichs Ungarn.*].

Scheiber zitiert die epidemiologischen Feststellungen von Purjesz: Fast alle Fälle betreffen nur die rumänische Bevölkerung, alle Kranken ernähren sich mit Maismehl, speziell im Kolozser [*Klausenburger*] Komitat trat diese Krankheit heuer zum ersten Male bei den Leuten auf, die sich bis zum heurigen Jahre stets mit Weizen- und Kornmehl nährten und nur heuer, nach einer dreijährigen Missernte, genötigt waren, hauptsächlich oder ausschließlich Maismehl zu verwenden.

Das Auftreten der meisten Pellagrafälle in Ungarn bei der Volksgruppe der Walachen lenkte die Aufmerksamkeit auf Rumänien. [*Als Walachen wird seit dem 10. Jahrhundert die bodenständige Bevölkerung von Walachei und Moldau bzw. des 1862 vereinigten Rumänien bezeichnet.*]

Was die ersten bekannten Pellagrafälle in Rumänien betrifft, besteht eine Unsicherheit. Das Zitat von Théophile-Victor-Jean-Baptiste Roussel (1816–1903), dass „Prof. [*Friedrich Wilhelm Felix von*] Bärensprung (1822–1864) im Jahre 1830 die ersten wohlconstatierten Fälle von Pellagra in Rumänien beobachtet“ habe, passt zeitlich nicht, da Bärensprung zu diesem Zeitpunkt erst acht Jahre alt gewesen ist. Verlässlich erscheint erst Scheibers Bericht, „im Jahre 1846 habe Dr. Alexander Theodori, Primararzt des Spitals in Rọman (Moldau), die ersten Fälle von Pellagra in diesem Spitale behandelt“. Als Scheiber „im Jahre 1862 als Spitalsarzt nach Jassy kam, kannten bereits alle Aerzte Rumäniens diese Krankheit, und man konnte in fast jedem der dortigen Spitäler und Irrenanstalten einzelne Fälle derselben beobachten“.

Die Ernährung der walachischen Bauern Rumäniens beschränkte sich im Winter fast nur auf Maismehl, das in Wasser zu einem dicken Brei gekocht wurde, der entweder pur oder mit Topfen vermengt oder in Form von in Milch eingetauchten Bissen gegessen wird. Dazu kamen noch Zwiebeln und Knoblauch. Im Sommer gab es schlechtes Obst, rohe Gurken und Salatblätter, nur mit Salz bestreut, ohne weitere Zubereitung. Die Walachen Siebenbürgens hatten ein besseres Leben, da sie bei Mangel in den dortigen ungarischen und deutschen Städten Arbeit fanden. Durch die nachbarliche Berührung hatten sie auch eine bessere Bearbeitung der Felder und Behandlung des Mais, den sie besser reifen und dann in Scheunen trocknen ließen. Die rumänischen Walachen dagegen bewahrten den nicht ausgereiften Mais in Gruben, wo er dumpf wurde [70].

## Maßnahmen gegen die Pellagra

Eine der Voraussetzungen für Bekämpfungsmaßnahmen von Volkskrankheiten ist deren Kenntnis bei den dazu berufenen Behörden. So war auch die Pellagra in der kurzen Zeit von 1888 bis 1894 in der Liste der anzeigepflichtigen [*heute: meldepflichtigen*] übertragbaren Krankheiten enthalten [64]. Das Sanitätsdepartement des k.k. Ministeriums des Innern meinte aber 1908 in seiner Stellungnahme im Herrenhaus, die Pellagra gehöre zu den „Krankheiten, welche zwar im strengsten Wortsinne vielleicht nicht als infektiös anzusehen, hinsichtlich der Maßnahmen aber gleich solchen zu behandeln sind“ [71].

Tatsächlich wurden aber im Allgemeinen die Erhebungen über das Vorkommen der Pellagra nicht auf das ganze Reich ausgedehnt, sondern auf der Ebene der Provinzen oder noch kleinerer Bereiche gepflogen. Entsprechend wurden auch die Maßnahmen meist für die betroffenen Provinzen angeordnet, wo die Durchführung auch auf bestimmte Bezirkshauptmannschaften beschränkt sein konnte.

**Figure 10:**
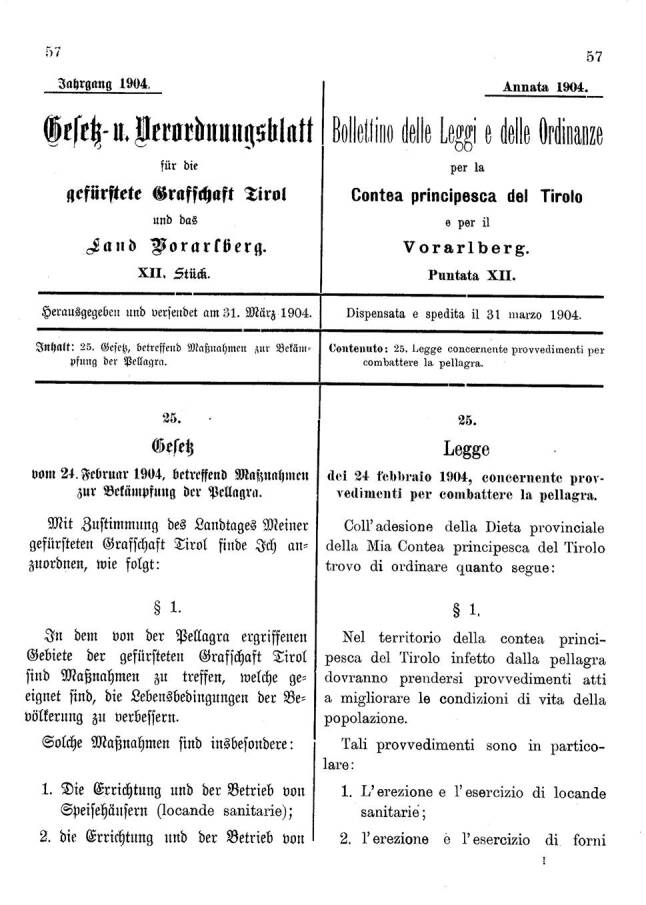

Eine gute Übersicht über notwendige Arbeiten gibt das im Gesetz- und Verordnungsblatt für die gefürstete Grafschaft Tirol und das Land Vorarlberg in deutscher und italienischer Sprache kundgemachte „Gesetz vom 24. Februar 1904, betreffend Maßnahmen zur Bekämpfung der Pellagra“ [72]. Die demokratische Genese des Gesetzes geht aus der Einleitung hervor, die lautet: „Mit Zustimmung des Landtages Meiner gefürsteten Grafschaft Tirol finde Ich anzuordnen, wie folgt“. Im Gesetz werden Maßnahmen für die von der Pellagra ergriffenen Gebiete Tirols angegeben, die „geeignet sind, die Lebensbedingungen der Bevölkerung zu verbessern“ (Abb. 10).

Der § 1 listet die sofort nach Kundmachung des Gesetzes durchzuführenden Maßnahmen auf; da diese nur demonstrativ angegeben sind, können sie bei Notwendigkeit auch ergänzt werden. Nämlich:

1. die Errichtung und der Betrieb von Speisehäusern (locande sanitarie);
2. die Errichtung und der Betrieb von Maistrockenöfen und Maislagerhäusern;
3. der Betrieb von Maisverkaufsmagazinen, in welchen gesunder Mais und Maisprodukte an die Bevölkerung abgegeben und gegen verdorbene oder minderwertige Ware eingetauscht werden;
4. Förderung von Brotbäckereien, welche von den Gemeinden in eigener Regie betrieben werden;
5. die Errichtung und Erhaltung von Pellagraheilanstalten (Pellagrosarien) und von Notspitälern für Pellagrakranke;
6. die Förderung der Niederlassung von Ärzten in solchen von der Pellagra ergriffenen Gemeinden, welche einer entsprechenden ärztlichen Hilfe entbehren;
7. die Belehrung der Bevölkerung über das Wesen der Pellagrakrankheit und über die Mittel zur Bekämpfung derselben;
8. die Organisation einer Pellagrastatistik;
9. die Ausschreibung und Zuerkennung von Preisen für wissenschaftliche Arbeiten und hervorragende Leistungen auf dem Gebiete der Pellagraforschung und Pellagrabekämpfung;
10. die Förderung des landwirtschaftlichen Betriebes, industrieller Unternehmungen, öffentlicher gemeinnütziger Arbeiten und Bauten im Pellagragebiete.

Weitere Paragrafen gaben die Grundlagen für die Durchführung der als notwendig erachteten Maßnahmen, darunter Folgendes:

Zur Bestreitung der erwachsenden Kosten ist ein eigener Pellagrafonds zu errichten, der durch Beiträge des Staates und des Landes sowie durch sonstige Zuflüsse gebildet wird.

Als beratendes und begutachtendes Organ wird in der Landeshauptstadt Innsbruck eine Pellagrakommission unter Vorsitz des Statthalters mit zwölf in ihrer Funktion festgelegten Mitgliedern sowie allfälligen beratenden außerordentlichen Mitgliedern eingesetzt.

Die politischen Behörden erster Instanz, also Bezirkshauptmannschaften und Magistrate der Statutarstädte, sind zur Mitwirkung bei der Durchführung dieses Gesetzes berufen. Die Gemeindevorsteher (Bürgermeister) sind verpflichtet, die politischen Behörden bei der Ausführung des Gesetzes zu unterstützen.

Die Gemeindeärzte sind verpflichtet, innerhalb ihres Amtssprengels, über Verlangen der politischen Behörden bei der Durchführung dieses Gesetzes, und zwar insbesondere bei der Überwachung der im § 1, Punkt 1 bis 5 bezeichneten Anstalten mitzuwirken. Die Gemeindeärzte sind ferner verpflichtet, alle ihnen zur Kenntnis gelangenden Fälle von Erkrankung oder Tod an Pellagra der politischen Behörde anzuzeigen.

Die technische Untersuchung von Mais und Lebensmitteln, insofern sich diese zur Bekämpfung der Pellagra nötig erweist, wird im Sinne des Lebensmittelgesetzes von der landwirtschaftlichen Versuchsstation in San Michele an der Etsch [*Südtirol*] auf Kosten des Pellagrafonds vorgenommen.

**Figure 11:**
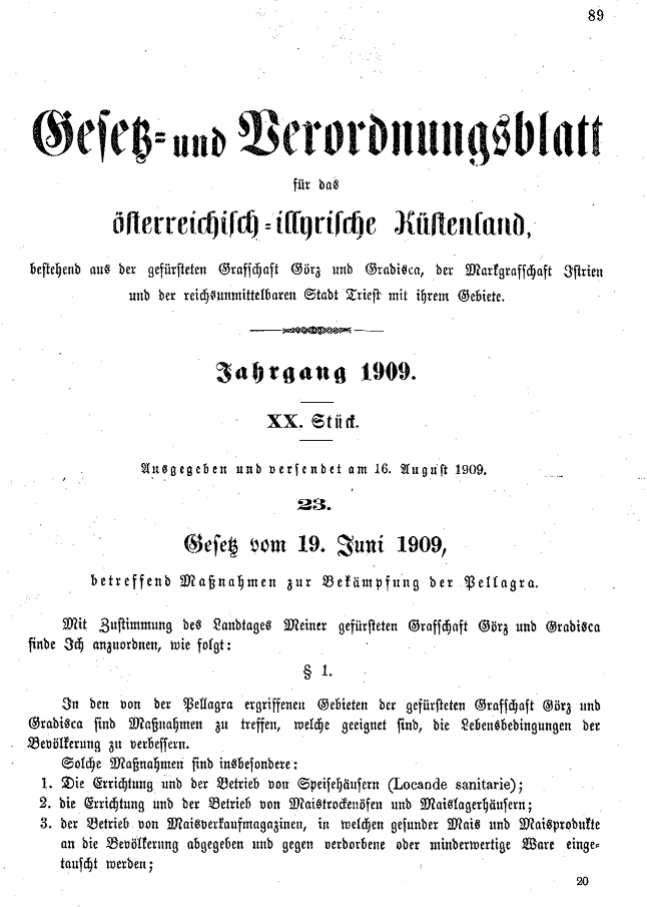

Gleiche Texte wie im Tiroler Gesetz wurden in den Gesetzen vom 19. Juni 1909 für die gefürstete Grafschaft Görz und Gradisca [73] und vom 1. Jänner 1911 für das Herzogtum Bukowina [74] verwendet. Nur war in Letzterem an Stelle des für die Durchführung der Maßnahmen verantwortlichen Statthalters der Landespräsident genannt, und für die Maisuntersuchung wurden Anstalten in Görz bzw. in Czernowitz bestimmt (Abb. 11).

Wenige Monate vor Ausbruch des 1. Weltkriegs berichtete der k.k. Landes-Sanitätsinspektor [*entspricht heute dem Sanitätsdirektor eines österr. Bundeslandes*] und Pellagrainspektor in Innsbruck, Ettore Weiß, im Detail über Durchführung und Ergebnisse der Pellagrabekämpfung in Südtirol [62]. Der Bericht hielt sich an die Reihenfolge der Paragrafen.

Die Speisehäuser für Arbeiter, Landbevölkerung und Schulkinder haben sich bewährt. Die in zehn Gemeinden gebauten für den Mais bestimmten Trockenöfen wurden jedoch von der Bevölkerung vorwiegend zweckentfremdet für die Abtötung der Seidenraupen in den Kokons verwendet. Dadurch waren die Seidenkokons, oft die einzige Einnahmequelle der Landbevölkerung, länger lagerbar bis zum Zeitpunkt höherer Verkaufspreise.

Maismagazine wurden nicht errichtet, da man eine Erhöhung der Ausgaben des Pellagrafonds für den im Gesetz vorgesehenen Umtausch von schlechtem Mais gegen einwandfreien vermeiden wollte. Man befürchtete nämlich, dass die Bevölkerung vermehrt Mais in den dafür nicht geeigneten Bereichen anbauen würde, um dadurch mehr minderwertigen Mais als bis dahin zum Umtausch bringen zu können.

Gemeindeeigene Brotbäckereien waren nicht administrierbar. Es wurden aber größere zentrale Anlagen in einigen Städten, zum Teil mit Teigwarenerzeugung, den sanitär- und gewerbehygienischen Anforderungen entsprechend eingerichtet. In diesen durften maishaltige Mehle nicht verwendet werden, sondern nur die übrigen Mischungen zur Verarbeitung gelangen. Im Jahr 1911 gab es bereits in acht Orten solche Bäckereien, die mit Kosten von 926.000 K [*Kronen*] in 58 Gemeinden über 67.000 Einwohner versorgten.

Als moderne Pellagraheilanstalt wurde das 1898 in Rovereto in einem Landhaus gegründete Pellagrosarium so ausgebaut, dass zuletzt jährlich 149 bis 168 Kranke aufgenommen werden konnten. Die Behandlung bestand dem Wissensstand entsprechend fast ausschließlich in Umstellung der Diät und körperlicher Kräftigung.

Zur Förderung der ärztlichen Versorgung wurden zwei Gemeindeärzteposten neu systemisiert, und weitere sieben Gemeinden erhielten Zuschüsse zur Bestreitung der Kosten für den Gemeindearzt von zwischen 250 und 2000 K jährlich.

Für die Belehrung der Bevölkerung hielten der Pellagrainspektor, die Amts- und die Gemeindeärzte besonders in den Anfangsjahren zahlreiche volkstümliche Vorträge über Wesen, Verhütung und Bekämpfung der Pellagra. Diese wurden ergänzt durch großflächige Verteilung von Flugblättern und eines Büchleins. Die sachkundig unterrichteten Schullehrer gaben ihre Kenntnisse in den Schulen weiter und auch der Klerus beteiligte sich auf Weisung des Trienter Fürstbischofs. Ferner wurden Mustergärten eingerichtet, um der Bevölkerung zu zeigen, dass der Anbau anderer Zerealien mit wenig Mühe und Aufwand bedeutend größere Vorteile bietet als der Maisanbau.

Die Durchführung der vom Gesetz geforderten Pellagrastatistik wurde durch mehrere Faktoren behindert. Es waren dies einerseits die unterschiedlichen Meldungen wegen der Vielfältigkeit der Pellagrasymptomatik und andererseits die unüberwindliche Scheu der Pellagrösen, ihre Krankheit zu zeigen, weil dies bei der bäuerlichen Bevölkerung als gleichbedeutend mit geisteskrank und dem Selbstmord verfallen galt. Weiß musste zugeben, dass somit die gesammelten Einzeldaten nur als Gesamteindruck zu werten sind, der den Stand der Epidemie vergleichsweise zu beurteilen ermöglicht. So sah man ab dem Jahr 1900 eine steigende, jedoch schwankende Tendenz mit dem Höhepunkt von 8053 Fällen im Jahr 1904. Interessant ist die Feststellung, dass die Spitzen der Mortalitätskurve in die Jahre von verschlechterten Ernährungsverhältnissen der bäuerlichen Bevölkerung durch Missernten oder andere ungünstige Vorkommnisse fallen.

Für die Fortbildung der Ärzte wurden in mehreren Jahren drei- bis viertägige Lehrkurse über Pellagra von Professoren der Universitäten Innsbruck, Pavia und Wien gehalten.

Von den Preisen für hervorragende wissenschaftliche Leistungen seien zwei genannt. Vom Innsbrucker Professors Ludwig Merk erschien 1909 in deutscher, italienischer und französischer Sprache das Lehrbuch über „Die Hauterscheinungen der Pellagra“. Exemplare der italienischen Ausgabe verteilte die Statthalterei an Gemeindeärzte des Pellagragebietes. Direktor Josef Schindler von der landwirtschaftlichen Versuchsstation in San Michele verfasste auf Anregung der Statthalterei die für die Maiskontrolle unentbehrliche „Anleitung zur Beurteilung des Mais und seiner Mahlprodukte als Nahrungsmittel“.

Bei der Förderung der landwirtschaftlichen Verhältnisse stand die Absicht der Auflassung der Maiskultur im Vordergrund. Maßnahmen dafür waren Prämien für die Umwandlung der Maisfelder in Wiesen oder anders bebaute Felder, Förderung der Viehzucht, Gratis- oder verbilligte Abgabe von Sämereien und Kunstdünger. Der Gebirgsbevölkerung wurde durch Verbesserung der Alm- und Milchwirtschaft geholfen.

Die Förderung der Industrie im südlichen Teil Südtirols, die kaum existierte, ist leider fehlgeschlagen. Weiß erklärte dies damit, dass zu deren Einführung „ein gewisser Schlag von Leuten mit gewissen Eigenschaften, unter welchen Ausdauer, Geduld und peinliche Reinheit voranstehen, Eigenschaften, welche in der Bevölkerung Südtirols erst langsam großgezogen werden müssen“ nötig ist.

In Österreich gab es im Gegensatz zu Italien kein Gesetz zur Maiskontrolle. Es wurden lediglich die Behörden erster Instanz über aus Ungarn und dem Ausland die Grenze überschreitende Sendungen von Mais und Maismehl informiert. Mit deren Kontrollen an den Bestimmungsorten waren ausschließlich in der Versuchsstation in San Michele dafür ausgebildete Amtsärzte betraut. Die bei der summarischen Untersuchung von ihnen beanstandeten Produkte wurden dann in dieser Anstalt nach verschiedenen Kriterien untersucht [75]. Die einheimischen Produkte waren größtenteils minderer Qualität und fast durchwegs in nicht ganz reifem Zustand gewonnen.

Weiß beschloss seinen Bericht mit der Feststellung, dass die Pellagra 1914 in Südtirol sowohl in der Zahl als auch in der Schwere im Abnehmen begriffen war. Die Ursache dafür sah er in der Besserung der Ernährungsverhältnisse der bäuerlichen Bevölkerung nach Abgehen vom ausschließlichen Maisgenuss und Verwendung der durch die Kontrolle nun besseren käuflichen Maismehle. Er meinte, dass dadurch „ein schädliches oder nachteilig wirkendes Agens durch die reichlicheren und zweckmäßigeren Zutaten der gegenwärtigen bäuerlichen Mahlzeiten neutralisiert, paralysiert wird“.

Der Tiroler Sanitätsreferent Ettore Weiß kommt 1914 also zu dem Schluss [40]: „Es mögen getrost die eingeführten Bekämpfungsmaßnahmen, welche die drei Hauptzwecke verfolgen 1. Hebung der wirtschaftlichen Lage, 2. Assanierung der allgemein hygienischen Verhältnisse und 3. Besserung der Ernährungsverhältnisse unter Ausschluß des Maises und ganz speziell des verdorbenen Maises fortgesetzt werden, denn dieselben haben, ob direkt oder indirekt, mag vorläufig dahin gestellt werden, eine ausgesprochene, überall und einstimmig hervorgehobene quantitative und qualitative Abnahme der Pellagrafälle zur Folge“. „Die Aufklärung der Bevölkerung über die Gefahren des Maiskonsums, welche auch zu den Pellagrabekämpfungsmaßnahmen zählt, hat ja vielleicht den Maiskonsum und die Maisbebauung bisher nicht in dem Maße eingeschränkt, daß man daraus das Abflauen der Intensität der Pellagraendemie vollständig erklären könnte, aber es ist sehr wohl möglich und meiner [*seiner*] Ansicht nach wahrscheinlich, daß die dadurch erzielte größere Sorgfalt in der Aufbewahrung des Mehls, in der Bereitung und Aufbewahrung der Polenta, besonders die Abnahme der Sitte, diese Polenta in Vorrat zu kochen und kalt aufzubewahren, zu den günstigen Resultaten vieles beigetragen hat“. Weiß sagt weiters, dass „in Südtirol seit dem Jahre 1905 die Pellagrafälle nicht nur ziffernmäßig ständig abnehmen, sondern auch, daß die früher nicht gar so seltenen schweren Formen der Pellagra jetzt förmlich Raritäten geworden sind und daß die Auffindung eines typischen Pellagraerythems, selbst dort, wo solche noch vor sieben bis acht Jahren verhältnismäßig häufig waren, heute kaum gelingt“.

Bezüglich der Verhältnisse in der Provinz Küstenland bemerkte Antonio Pontoni aus Görz im Jahr 1913 [76], dass „das Pellagrosarium, wie es heute im Küstenland organisiert ist, eigentlich keine richtige Existenzberechtigung hat. Die schweren Fälle können dort nicht interniert werden, die gehören der Irrenanstalt oder dem Krankenhaus. Die Fälle werden weder durch Pellargrosarien, weder durch Forni rurali [*ländliche Backöfen*] noch durch Locarde sanitarie [*Speisehäuser*], sondern werden geheilt wie andere schwere Geisseln der Menschheit durch die Lösung des großen sozialen Problems“.

Im selben Jahr bestätigte Luigi Devoto (1864–1936) in Mailand [77]: „Die Pellagra ist aus Piemont verschwunden und im Trentino [*italienisch-sprachliches Südtirol*] am Aussterbeetat.“ Er stellte fest, „dass in der Lombardei die Pellagra verschwindet, weil der Maisgenuss eingeschränkt wird, weil der Genuss des verdorbenen Maises ausgeschaltet wird, weil die Aufklärung und der ökonomische Wohlstand fortschreiten, weil die gemischte Ernährungsweise ihren Einzug beim Bauern hält und endlich, weil der Kampf gegen die Pellagra allgemein geworden ist.“

In der Bukowina kam erst später Bewegung in die Bekämpfung der Pellagra. Die lokalen in Czernowitz erscheinenden Zeitungen [64, 70, 78] machten 1909 und 1910 durch ihre Berichte offenbar Druck auf den Landtag und wohl auch auf das griechisch-orientalische Konsistorium. Es wurde berichtet, dass in den Jahren 1905–1909 zwar Brotbäckereien zur Ausgabe von Brot, Speck und Salz errichtet worden sind. Auch wurden Gelder von Staat, Land und griechisch-orientalischem Religionsfonds aufgewendet für die Anschaffung von Kunstdünger, Ziegen und Schafen für die arme Bevölkerung. Doch hält man die Bekämpfung der Pellagra trotzdem nicht für möglich, solange die Bevölkerung an den ausschließlichen Genuss von Mais gewöhnt ist. Eine ganz besondere Bedeutung wird der Beseitigung, aber zumindest einer weitgehenden Einschränkung des Fastens bei der bäuerlichen Bevölkerung beigemessen. Speziell bei der griechisch-orientalischen Bevölkerung ist das vier Fünftel des Jahres umfassende Fasten der Hauptgrund ihrer Unterernährung. Es ist also im Einvernehmen mit dem Konsistorium das Fasten einzuschränken. [*Zur angegebenen Abgabe von Salz* [79]*: Auf Anregung des Abgeordnetenhauses des Reichsrates holte das Ministerium des Innern 1910 die Meinung des k.k. Obersten Sanitätsrates ein, ob Kochsalz ein Mittel gegen die Pellagra ist. Dieser hielt dies nicht für sichergestellt, meinte aber, dass eine verbilligte Abgabe ein Mittel zur Armenunterstützung sein kann.*]

## Schlusswort

Die Pellagra war in manchen Gegenden und zu verschiedenen Zeiten eine mit Letalität behaftete und die Volksgesundheit bedrohende Krankheit. Seit 1771 im Kaiserreich Österreich in der Lombardei bekannt, wurde sie danach vorwiegend in den Kronländern Tirol, Küstenland und Bukowina angetroffen. Überall dort war die Pellagra eine Geißel armer Bevölkerungsgruppen, deren Ernährung fast ausschließlich auf schlechtem Mais beruhte.

War also die Armut die Ursache der Krankheit? Schon bald erschien ein Zusammenhang mit der Ernährung durch Mais möglich, vielleicht auch sicher? Die Krankheit erwies sich als nicht kontagiös, war aber vielleicht durch Erreger verursacht, oder durch giftige Produkte? War sie gar eine allergische Reaktion der Betroffenen auf die Sonnenbestrahlung oder auf Bestandteile des Maises? War aber vielleicht, was immer wahrscheinlicher wurde, die einseitige Ernährung mit Mais am Entstehen der Pellagra Schuld?

Viele Möglichkeiten, aber welche stimmte? Vielleicht keine?

Die Lösung brachte die Feststellung Mitte der 1940er-Jahre in den USA, dass der Mais nicht der Träger eines schädlichen Agens war, sondern im Gegenteil, ihm fehlte bei der außerhalb seiner Heimat Mittelamerika üblichen Zubereitung die Freisetzung eines wichtigen Vitamins, des Niacins, das bei gemischter Kost in genügender Menge aufgenommen wird.

## Anhang

### Biografische Daten

**Domenico Majocchi**, geb. am 5. August 1849 in Roccalvecce bei Rom. Nach erfolgreicher chirurgischer Tätigkeit in Rom Wechsel zur in Italien noch wenig entwickelten Dermatologie. Mangels lokaler Vorbilder richtete er sich nach den pathologisch-anatomischen Vorgaben des von ihm bewunderten Ferdinand von Hebra in Wien. 1880 bzw. 1892 wurde er Klinikchef in Parma bzw. in Bologna. Er beschrieb viele dermatologische Krankheiten, von denen zwei seinen Namen tragen. Seine doch wesentliche Bearbeitung der Pellagra wurde in biografischen Artikeln nicht erwähnt. Majocchi wurde 1924 emeritiert und starb am 07. März 1929 in Bologna.

**Giuseppe Cuboni**, geb. am 2. Feber 1852 in Modena. Studium der Medizin und Naturwissenschaften in Rom. 1877 bis 1881 Arbeit im Botanischen Garten in Rom, danach in der Weinbauschule in Conegliano, Provinz Treviso, also einer Pellagra-Gegend, als Professor für Naturwissenschaft (1881–1885) bez. Botanik und Pflanzenphysiologie (1886–1887) und 1887–1920 Direktor der Station für Pflanzenpathologie in Rom. Cuboni starb am 3. November 1920 in Rom.

**Edmund von Neusser**, geb. am 1. Dezember 1852 in Swoszowice im k.k. Kronland Galizien. Nach Abschluss des Studiums in Krakau und Wien 1877 tätig als Assistent bei Adalbert Duchek, Anton Drasche und Heinrich von Bamberger. 1888 Habilitation für Innere Medizin, 1889 Primarius an der Krankenanstalt Rudolfstiftung und von 1893 bis zu seinem Tod am 30. Juli 1912 in Fischau, Niederösterreich, war er Vorstand der Wiener 2. Medizinischen Universitätsklinik. Er galt als ausgezeichneter Kliniker, der jedoch wenig publizierte.

**Richard Paltauf**, geb. am 9. Feber 1858 in Judenburg in der Steiermark, Medizinstudium in Graz mit Promotion am Weihnachtstag 1880. Danach bis 1883 Assistent am dortigen Pathologisch-anatomischen Institut unter Hans Kundrat, unterbrochen durch Teilnahme am Insurrektionsfeldzug in Süddalmatien (Jänner – September 1882). Ab Ostern 1883 bei Kundrat nun in Wien, dort 1888 Habilitation für Pathologische Anatomie. Paltauf hatte großes Interesse an der neuen Wissenschaft der Bakteriologie; so bearbeitete er u. a. das Rhinosklerom, die Hauttuberkulose, die Diphtherie, die Hadernkrankheit, das Wechselfieber und die Pellagra. Nach seiner Ernennung zum a. o. Professor 1892 gründete er in der Wiener allgemeinen Poliklinik ein Histologisch-bakteriologisches Institut. 1893 wurde er Prosektor der Krankenanstalt Rudolfstiftung und Vorstand des Universitätsinstituts für Pathologische Histologie und Bakteriologie sowie Leiter der Anstalt für Wutschutzimpfungen. 1894 begann er die Produktion von Diphtherieheilserum in seiner Prosektur, woraus in der Folge die „Produktionsanstalt“ und schließlich das „Serotherapeutische Institut“ entstanden. Paltauf wurde 1900 o. Professor für allgemeine und experimentelle Pathologie in Wien; im 1. Weltkrieg war er als Generalstabsarzt Berater im K. u. k. Kriegsministerium. Richard Paltauf erlag einem Bronchuskarzinom am 21. April 1924 auf dem Semmering in Niederösterreich.

**Cesare Lombroso**, geb. am 6. November 1835 in Verona im Königreich Lombardo-Venetien im Kaisertum Österreich. Er studierte in Turin, Pavia und Wien. 1862 wurde er Professor der Psychiatrie in Pavia und später Professor der Gerichtsmedizin und Psychiatrie in Turin. Er befasste sich intensiv mit Geisteskrankheiten und Verbrechertum. Doch publizierte er auch 1872 über „Klinische und experimentelle Studien der Pellagra“ und 1880 „Über Gifte des Mais“. Lombroso starb am 19. Oktober 1909 in Turin.
